# Leadership practices of physical education teachers and student-related outcomes: a systematic mixed method review and analysis

**DOI:** 10.3389/fpsyg.2024.1442014

**Published:** 2024-11-01

**Authors:** Hyun-Duck Kim, Angelita Bautista Cruz

**Affiliations:** ^1^Department of Sport Marketing, Keimyung University, Daegu, Republic of Korea; ^2^Department of Physical Education, Keimyung University, Daegu, Republic of Korea

**Keywords:** teacher leadership, data mining, sports curriculum, autonomy-support behavior, motivation, student sex differences, regional differences, meta-analysis

## Abstract

**Introduction:**

This study systematically reviewed and analyzed both qualitative and quantitative studies that focused on the relationship between physical education (PE) teachers’ leadership and student outcomes using data mining and meta-analysis.

**Methods:**

Using the Scopus, PsycINFO, PubMed, and SPORTDiscus databases, text data from the included 150 relevant articles were examined through a text data mining approach. Quantitative and mixed-method studies were then further evaluated, yielding 49 articles eligible for inclusion in the meta-analysis.

**Results:**

Findings from the data-mining analysis using Leximancer revealed eight major themes associated with PE teacher leadership, emphasizing motivation, education, support, and teaching. Results from the meta-analysis using the Comprehensive Meta-Analysis software showed that autonomy-supporting behaviors showed strong positive effects on student satisfaction, motivation, competence, and engagement. Regional differences in research focus were observed, with health being less emphasized in Asian and European studies. Student sex emerged as a moderating factor.

**Conclusion:**

The study highlights the role of PE teacher leadership and the convergence/divergence among research findings. Moreover, applying the HDST approach to synthesize both qualitative and quantitative articles provided a deeper and more comprehensive understanding of leadership within the PE field.

## 1 Introduction

Leaders are regarded as important individuals in any organization, as they are generally described as those who influence or guide members or followers to accomplish goals. Leaders are designated in various organizations or institutions, such as businesses, governments, and educational institutions. In physical education (PE) for instance, teachers are delegated classroom leaders during PE class. They teach and demonstrate fundamental motor skills, sports, and other physical activities. They also educate students about health-related topics, such as stress management, nutrition, health conditioning, safety, and prevention. In addition to developing motor skills and fitness competencies, PE teachers also promote students’ life skills, including teamwork, persistence, and sportsmanship, through PE activities and programs (Cronin et al., 2023). To fulfill these teaching roles, effective teaching and leadership behaviors are crucial for teachers when conveying PE-related knowledge and skills to students. A teacher who demonstrates positive behaviors while delivering class lessons is likely to enhance student motivation, class participation, and perceptions of PE, and more importantly, encourage students to regularly engage in active and sustainable healthy lifestyles. In contrast, a teacher who lacks organization, delivers unstimulating lessons, and is indifferent to students’ needs, may negatively impact students’ attitudes (Cruz et al., 2021), feelings (Van den Berghe et al., 2015), and engagement in PE and other physical activities (Hassandra et al., 2003). Therefore, it is paramount for PE teachers to possess the appropriate teaching skills and leadership behaviors that foster positive experiences for students in PE, which in turn can influence the adoption of healthy active lifestyle. By doing so, PE teachers not only achieve the learning goals of PE but also effectively fulfill their roles as classroom leaders.

Currently, approximately 81% of adolescent students (11–17 years old) of the world’s population do not meet the required physical activity recommendations of the World Health Organization (WHO) (Guthold et al., 2020). This high level of physical inactivity in school-aged populations can lead to future adverse health-related repercussions (Katzmarzyk et al., 2022) unless opportunities to actively involve students in physical activities are implemented. Studies revealed that students’ motivational and emotional experiences in PE can promote either physical inactivity (Beltrán-Carrillo et al., 2012; Cardinal et al., 2013; Fierro-Suero et al., 2022) or participation in leisure time physical activity (PA) (Castillo et al., 2020; Chen et al., 2014; Fierro-Suero et al., 2022). In this case, given that PE teachers have direct contact with students during PE classes, they possess the capacity to create a classroom climate and learning culture that can affect students’ responses through their interactions when teaching PE curriculum content and physical activities. The way PE teachers instruct, inspire, support, and provide feedback can either negatively or positively affect students’ feelings, attitudes, and behaviors resulting from their PE experiences. Thus, it is crucial for PE teachers to acquire and demonstrate effective leadership traits, skills, and behaviors when interacting with students during PE class, as their teaching and leadership approaches and strategies can have an influential role in shaping students’ affective, cognitive, and behavioral outcomes. Physical education teachers who practice appropriate and positive leadership during class can effectively enhance student motivation to engage in PE and lifelong physical activity participation, thereby contributing to achieving PE objectives as well as supporting Sustainable Development Goals (e.g., SDG3 good health and well-being) (World Health Organization, 2018).

Although various approaches can be used to understand the influential role of PE teachers as leaders in education, the transformational leadership approach (Bass, 1998) has recently emerged as a promising framework. This framework is widely used to study leadership behaviors in occupational settings, but has recently been extended to the field of education and conceptualized as transformational teaching (Beauchamp and Morton, 2011). According to Beauchamp and Morton (2011), who advocate for using transformational leadership theory in the PE domain, effective leadership and teaching are quite similar because both involve the process of influencing others in a social context to achieve established goals. They also contend that while transformational teaching is grounded from transformational leadership, it also share similar theoretical concepts from several common teaching and behavior change models such as social learning theory (Bandura, 1977), self-determination theory (Deci and Ryan, 2012), and pygmalion effect (Rosenthal and Jacobson, 1968). Transformational teaching is described as inspiring, motivating, and building students’ confidence to achieve higher levels of functioning, as well as transcending the teacher’s own self-interests by exhibiting transformational behaviors. These transformational behaviors include individualized consideration, intellectual stimulation, inspirational motivation, and idealized influence. Individualized consideration refers to the degree to which a teacher supports students’ needs and desires and a concept which conforms with psychological need satisfaction within the self-determination theory (Beauchamp and Morton, 2011; Deci and Ryan, 2012). Intellectual stimulation is the degree to which a teacher encourages students to be creative, critical thinkers, and problem solvers and considered to offer a unique view in understanding the effects of transformational behaviors of teachers in physical education (Beauchamp and Morton, 2011). Inspirational motivation is the degree to which a teacher communicates task objectives clearly and meaningfully, which matches the idea of the Pygmalion effect that high and clear expectations can affect performance (Rosenthal and Jacobson, 1968). Idealized influence is the degree to which a teacher resonates with charisma and acts ethically and with integrity among students and a concept similar to role modeling within the social learning theory (Bandura, 1977). Showing these transformational leadership qualities and characteristics to students tends to create a teacher-student connection built on trust, respect, and loyalty, thereby inspiring students to achieve established individual and group class goals. Based on these assumptions, for students to enhance their motivation to participate in PE and their commitment to achieve the desired PE outcomes, PE teachers should demonstrate a high degree of transformational behaviors that consider students’ physical strengths and weaknesses, encourage the development of students’ creativity and higher-order cognition through sports and games, build students’ passion to pursue health and fitness objectives, and set a good example for students by maintaining a healthy weight and practicing active living and healthy behaviors. Therefore, finding the right balance of teaching behaviors that elicits positive outcomes in students is vital for becoming effective PE teachers.

Numerous studies have shown that PE teachers’ teaching behaviors can have a profound influence on student outcomes. It was found that PE teachers who provide positive verbal feedback (Koka, 2010; Koka and Hein, 2003), cultivate student autonomy (Van den Berghe et al., 2015; Lewis, 2014), display supportive teaching (Van Doren et al., 2021; Tilga et al., 2020) and practice democratic (Kokkonen et al., 2013; Koka, 2010), and transformational teaching behaviors (Verma et al., 2019; Castillo et al., 2020; Hernández-Martos et al., 2023) affected students’ positive affect (Beauchamp et al., 2010), satisfaction (Hsu et al., 2022), motivation (Koka and Hein, 2003; Castillo et al., 2020), and engagement (Van den Berghe et al., 2015; Verma et al., 2019) in PE.

In contrast, findings also revealed that PE teachers who demonstrate uncaring and controlling behaviors (Hein et al., 2015; Koka et al., 2019; Timken et al., 2019) can lead to students’ disengagement (Leo et al., 2022; Van den Berghe et al., 2015), demotivation (De Meyer et al., 2014; Koka et al., 2019), boredom (Sanchez-Oliva et al., 2014), and dissatisfaction (Van den Berghe et al., 2015) in PE classes, which in turn are related to decrease in class performance (Behzadnia et al., 2018) and even decreased intention to participate in physical activities outside of PE class (Behzadnia et al., 2018; Koka et al., 2019; Viksi and Tilga, 2022) due to the unfavorable experiences toward PE.

With existing studies on PE teachers’ teaching styles and behaviors, researchers have attempted to summarize qualitative (White et al., 2021) and quantitative studies (Guo et al., 2023; Lochbaum and Jean-Noel, 2016; Tao et al., 2022; Vasconcellos et al., 2020). For instance, a review of qualitative studies has revealed the emergence of various themes related to PE teachers. Specifically, PE teachers who are friendly, enthusiastic, and actively engaged with students during class positively influenced students’ intrinsic motivation and participation in PE. Moreover, PE teachers who provide students with novel tasks or different options in terms of activities and roles, emphasize fun, challenge, and teamwork, and personally involved in class lessons and activities with students resulted in students reporting enhanced motivation, perceived competence, self-efficacy, and PE participation (White et al., 2021). PE teachers who emphasize competition and performance scores, only pay attention to physically talented students, publicly choose teams without any student consultation, and allow students to perform individually in front of others have been found to undermine students’ motivation and perceived competence (White et al., 2021). On the other hand, findings from quantitative studies using a meta-analysis revealed that PE teachers who provided autonomy support instruction showed large effects on students’ motivation and positive emotions during PE and leisure time. However, the influence of PE teacher autonomy support on students’ physical activity motivation is weak (Lochbaum and Jean-Noel, 2016).

Indeed, a systematic review of qualitative and quantitative studies has provided better insights into the critical role of PE teachers’ behaviors in shaping students’ learning outcomes. However, because of the vast number of textual documents that were manually checked and evaluated during data synthesis in a previous qualitative review (White et al., 2021), it is likely that other concepts or themes related to PE teacher leadership and student outcomes were omitted and thereby remain open for further exploration. The use of a more sophisticated, automatic, unbiased, and faster approach to synthesizing and summarizing content from large collections of documents may provide new and more in-depth insights into the key concepts associated with this topic. Whereas previous meta-analyses often encountered large variations in student outcomes (Lochbaum and Jean-Noel, 2016; Vasconcellos et al., 2020), researchers tend to combine these variables, leading to potential challenges in understanding the nuances or distinct consequences of the phenomenon under investigation. Additionally, because meta-analysis studies are generally concerned with statistical values to calculate and summarize relevant variables, studies with or without insufficient quantitative data are often excluded (almost 90%) from the assessment process, thereby losing considerable information that may be vital for researchers. It would therefore be noteworthy to first explore the content of these related studies by systematically analyzing these documents to identify prospective distinct variables prior to summarizing the statistical data of quantitative studies. Thus, it is likely to draw better conclusions about the overall impact of PE teachers’ leadership styles and behaviors on students’ learning outcomes.

Recently, a novel mixed-method approach has been developed, called the Hybrid Data Synthesis Technique (HDST) (Kim and Cruz, 2022), which synthesizes relevant studies on a given topic and evaluates both lexical and numerical data via content analysis and meta-analysis. The advantage of the HDST approach is that researchers can gain an overall and more robust perspective on a certain topic by examining both qualitative and quantitative studies for potential concepts and themes using data mining and analyzing numerical information via meta-analysis. The outcomes are then used to determine whether the lexical and statistical findings converge or diverge from one another. With these summative findings, researchers and/or practitioners can gain more meaningful insights into the research topic being investigated, which can be potentially overlooked using conventional systematic review approaches and, in turn, provide appropriate practical and/or theoretical recommendations on how to solve a problem and/or advance a research field. For example, when using the HDST to examine the relationship between transformational leadership and well-being in the service industry, the authors found convergence between themes, concepts, and statistical results related to leadership and well-being. However, they also identified findings that were either unique or inconsistent with the results from separate qualitative and quantitative analyses, highlighting both the limitations of each research methodology and potential directions for future studies. Notably, they encourage other researchers to consider using this approach when exploring data from both qualitative and quantitative studies (Kim and Cruz, 2022). Hence, adopting the HDST approach in collecting, synthesizing, and evaluating both qualitative and quantitative studies on PE teacher leadership and student outcomes may reveal new themes and concepts associated with this research area coming from all available textual documents as well as determine the magnitude of the relationship between the predictor and outcome variables based on relevant statistical data, thereby offering a more robust and comprehensive understanding of the research landscape of leadership in PE in general and the impact of the leadership styles and behaviors of the PE teacher on various learning outcomes in particular.

Therefore, the purpose of this investigation was to systematically review and analyze both qualitative and quantitative studies that focused on the relationship between PE teachers’ leadership and student outcomes in the field of PE using the HDST approach (Kim and Cruz, 2022). The following research questions are explored in this study:

1. What major themes and concepts would emerge from the synthesized lexical data related to the leadership of PE teachers and student outcomes?
2. What major themes and concepts would emerge from the synthesized lexical data related to PE teachers’ leadership and student outcomes based on regional affiliations?
3. What is the overall effect size (ES) of the relationship between PE teacher leadership and pertinent student outcomes?
4. Will the leadership behaviors of PE teachers affect student outcomes differently for male and female students?
5. Will there be convergence or divergence between the results of qualitative and quantitative studies?

## 2 Method

This study employed a mixed-method approach to answer its research objectives, consistent with the critical appraisal methodologies proposed by Heyvaert et al. (2013) and Kim and Cruz (2022). The mixed-methods approach integrated both textual (qualitative) and numerical (quantitative) data, acknowledging the significance of combining these forms of evidence to produce thorough insights (Pluye and Hong, 2014; Schoonenboom and Johnson, 2017).

The methodological technique for data synthesis in this study was delineated into two separate phases. The initial phase involved the collection and preliminary analysis of both qualitative and quantitative data, ensuring that each data type was meticulously assessed for its possible themes and concepts. The second phase, adhering to the recommendations of Kim and Cruz (2022), assessed the effect size scores concerning potential linkages among underlying factors and moderators for a comprehensive interpretation. The Hybrid Data Synthesis Technique (HDST) combines the strengths of narrative (qualitative) and numerical (quantitative) data, adhering to the guidelines established by Pluye and Hong (2014) and Kim and Cruz (2022) to leverage the “power of stories and numbers” in formulating robust conclusions.

### 2.1 Phase 1: systematic review and data mining

#### 2.1.1 Phase 1 text-data source selection

For the initial analysis of Phase 1, the data sources utilized were the Scopus, PsycINFO, PubMed, and SPORTDiscus databases. The authors simultaneously performed the initial search across these databases using a combination of interconnected keywords, including terms like “physical education teacher,” “leadership,” “leadership styles,” “teacher behavior,” “student outcomes,” “student satisfaction,” “student engagement,” and “learning outcomes” Inclusion criteria for studies required that they: (1) written in English and available in full text, (2) focus on physical education context and its influence over students at various levels, and (3) be published in peer-reviewed journals prior to June 3, 2023, with the relevant scope range regarding the keywords listed above.

**Figure 1:**
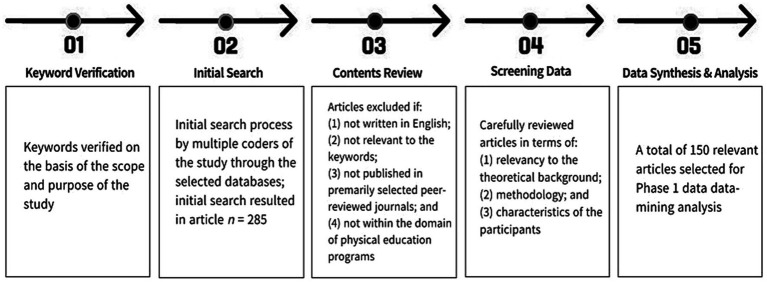
Procedural details of text-data coding and analysis.

The initial literature search yielded 19,597 articles. After further filtering according to the specified inclusion parameters and removing duplicates, 285 published articles were eligible for additional screening. The authors conducted individual screening searches and obtained pertinent documents by carefully evaluating the content based on predetermined inclusion criteria. Eligible documents were subjected to a rigorous review process and were subsequently excluded based on the following criteria: (1) not within the context of physical education (*n* = 118), (2) unavailability of the full text for each article (*n* = 3), (3) non research articles (*n* = 6), (4) physical education topic unrelated to teacher leadership/student outcomes (*n* = 4), or (5) dispute regarding the article’s relevance and selection (*n* = 4). The authors extensively examined whether an article met the selection criteria, but failed to reach a consensus of 80%. Of the initial collection of articles (*n* = 285), 52.6% (*n* = 150) were deemed pertinent for Phase 1 of the analysis, as determined by the selection criteria and by incorporating both qualitative and quantitative approaches. To prepare for additional data synthesis and analysis, the incorporated articles were converted into an analyzable format. Additional information regarding the techniques used for synthesizing and analyzing the data is shown in Figure 1.

#### 2.1.2 Data-mining analysis

The text data from the initial set of 150 articles were examined through a text data mining approach employing Leximancer (version 5.0; Leximancer Pty Ltd). This software facilitates the visualization of text data by validating and presenting thematic and conceptual maps. It can uncover and highlight interconnections or groupings of themes and concepts. An essential advantage of this data mining technique is its proficiency in visualizing the central topics within text data. This visualization aids in comprehending the inherent structure of the text data (Angus et al., 2013; Hyndman and Pill, 2018). Leximancer is widely recognized as a text analytic tool that automatically generates a thesaurus of words and phrases based on contextual similarities as well as semantic and relational extraction (Thomas, 2014).

Based on the theoretical framework of the Bayesian decision theory (Bülthoff and Yuille, 1996), this analytical tool generates a graphical representation using its conceptual and thematic mapping algorithms. Forming equivalent thematic and conceptual networks (Hyndman and Pill, 2018; Thomas, 2014), the concept map of Leximancer depicts the relative co-occurrence of words and phrases. Leximancer generates bubble-shaped clusters representing “concepts” that have been transformed or classified into “themes” according to their associated keywords (Angus et al., 2013; Thomas, 2014), after the analysis of the data. To comprehend Leximancer’s concept map, the following characteristics must be considered. (1) The clusters are depicted by colors that indicate the degree of importance for each subject. Warm shades such as red and orange signal a higher level of connectivity to the theme, while cool colors like blue and green signify a lesser level of connectedness to the theme. Further details can be found in Leximancer user guide release 5.0, where the magnitude of a theme’s circle signifies its degree of interconnection within the program’s concept map (Lemon and Hayes, 2020).

### 2.2 Phase 2: meta-analytic review

#### 2.2.1 Phase 2 coding data for meta-analytic review

**Figure 2:**
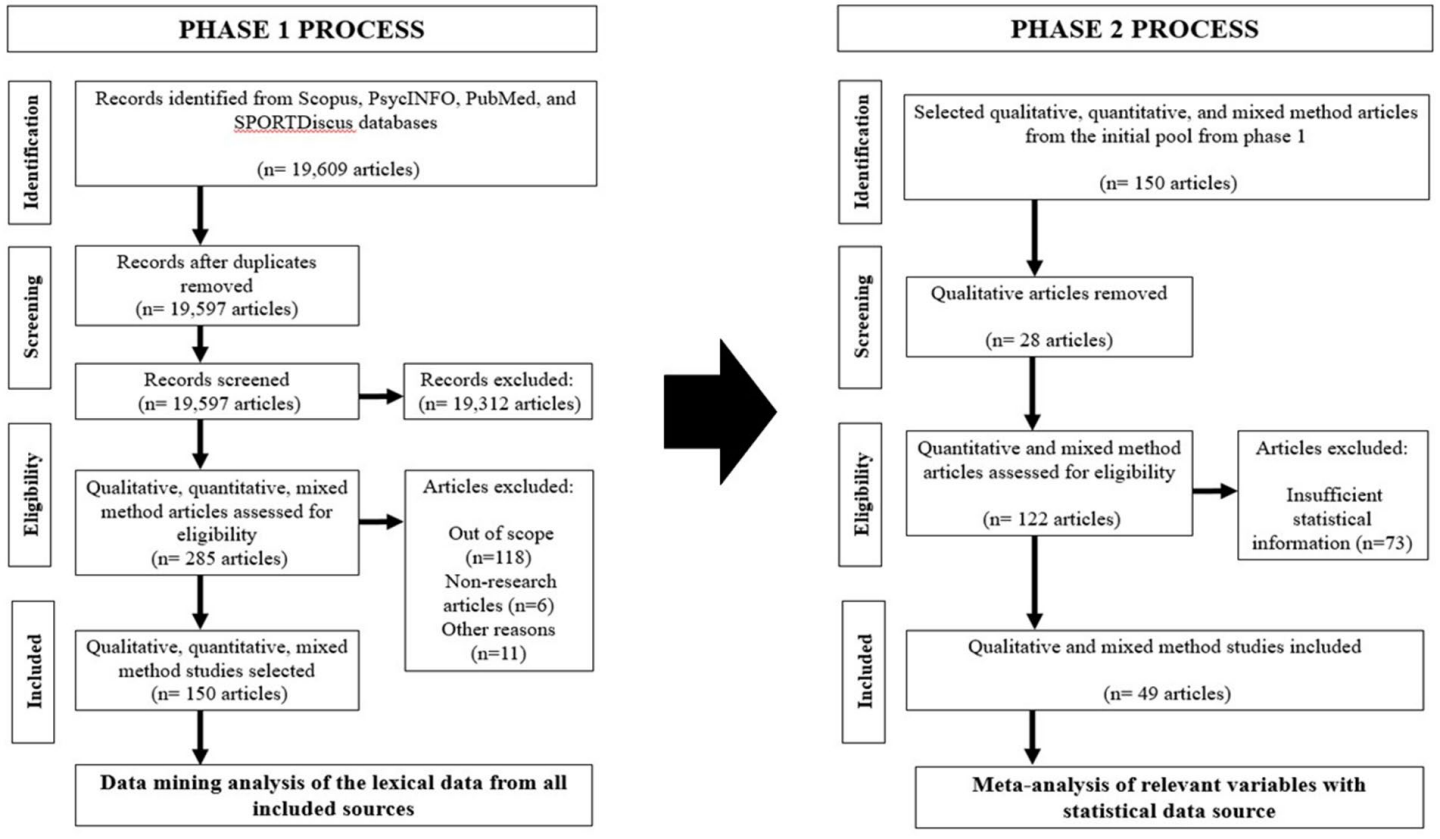
Flow diagram of the Hybrid Data Synthesis Technique for phase 1 and phase 2 processes.

A comprehensive examination of the textual data obtained in phase 1 was conducted in phase 2 through a meta-analysis of the selected articles. During phase 2 of the analysis, we adhered to the fundamental procedures recommended by the Preferred Reporting Items for Meta-Analysis (PRISMA) standards (Panic et al., 2013). Out of the original set of 150 articles, studies employing a qualitative design were automatically eliminated (28 studies), whereas the remaining mixed-method and quantitative studies underwent further evaluation. Of the 122 studies examined, 49 with a combined sample size of 13,779 were considered eligible for inclusion in the meta-analysis because these studies contained correlation matrices with adequate statistical information to compute ES (Figure 2). The selected articles were grouped according to gender ratios (i.e., the proportion of different sexes in the PE Programs) to determine the potential moderating effect of student gender on the relationship between PE teachers and student outcomes, as proposed in a previous study (Guo et al., 2023). The specific processes of Phases 1 and 2 are outlined in Figure 2.

Possible publication bias for the studies accepted in this study were assessed by the funnel plot of Hedges’s g values based on their standard errors. It is important to gain insights into potential presence of publication bias for further meta analytic analyses. Studies accepted with larger sample sizes (i.e., therefore more precise estimates) tend to be plotted symmetrically and also toward the top of the funnel plot graph, indicating no publication bias (see Supplementary Figure 1-funnel plot). Based on the funnel plot of PAS and PO variables, the distribution of plot showed symmetry of points on both sides, suggesting no indication of publication bias. Moreover, the Hedges’s g values was.5057, *p* > 0.05 which also confirms no publication bias.

A statistical model utilizing random effects was chosen for the analysis of the data (Petitti, 2001) based on the assumption of heterogeneity. A random-effects meta-analysis was conducted according to the guidelines provided by Hunter and Schmidt (2004).

Comprehensive meta-analysis software (CMA) was used to compute pertinent statistical data, including sample sizes and correlations, acquired from quantitative studies. These data were used to estimate 95% confidence intervals and the extent of certainty (Borenstein et al., 2009). A value of ES equal to or greater than 0.5 is considered to be a large effect size, while a value between 0.30 and.49 is considered to be a moderate effect size. A value less than 0.30 is considered to be a small effect size (Fritz et al., 2012; Cohen, 1988).

## 3 Results

### 3.1 General results

Overall, the included articles related to PE leadership were published from 2003 to 2023. Majority (84%) were conducted from 2011 to 2023 and with the highest number of studies observed in 2021 (23 articles). In terms of region, 47% of the articles were conducted in Europe, followed by North America (25%), Asia (17%), and Oceania and others (11%). The research method used was mostly quantitative studies (71%), while the remainder employed qualitative methods (19%) and mixed methods (10%). The summary information of all articles can be found in the Supplementary Table-summary of articles.

### 3.2 Results of the data-mining analysis

The analyses for this research were conducted in two distinct phases, as described in the methodology section. Using data mining technology, the initial phase entailed an examination of selected articles concerning PE teacher leadership and student-related outcomes. The objective of this procedure is to authenticate common themes and ideas extracted from the textual data of the selected articles.

The graphical representation generated by Leximancer data-mining software is known as an “concept/cluster map,” which illustrates the concepts and themes prevalent in a given text data source. The relevance of a theme or concept as well as the degree of mathematical interconnection among words is discernibly indicated on the concept map through variations in the size and color of the words (Thomas, 2014). The ability to generate conceptual maps throughout the production of meaningful outputs is the most distinctive characteristic of this analytical instrument. In terms of the capacity to handle substantial amounts of data and resistance to human bias, this characteristic establishes this analytical instrument is more effective than conventional qualitative analysis approaches (Sotiriadou et al., 2014). The key characteristics of the Leximancer conceptual map include the following: (1) colors, which represent the significance of each theme (e.g., the least connected theme is purple, and the most significant is red) and (2) the magnitude of the theme, which signifies the degree to which a concept appears to cluster with other concepts in a single text-mining analysis. The terms “themes” are used to denote clusters of concepts that range in temperature from warm (red, orange) to cold (blue, green).

**Figure 3:**
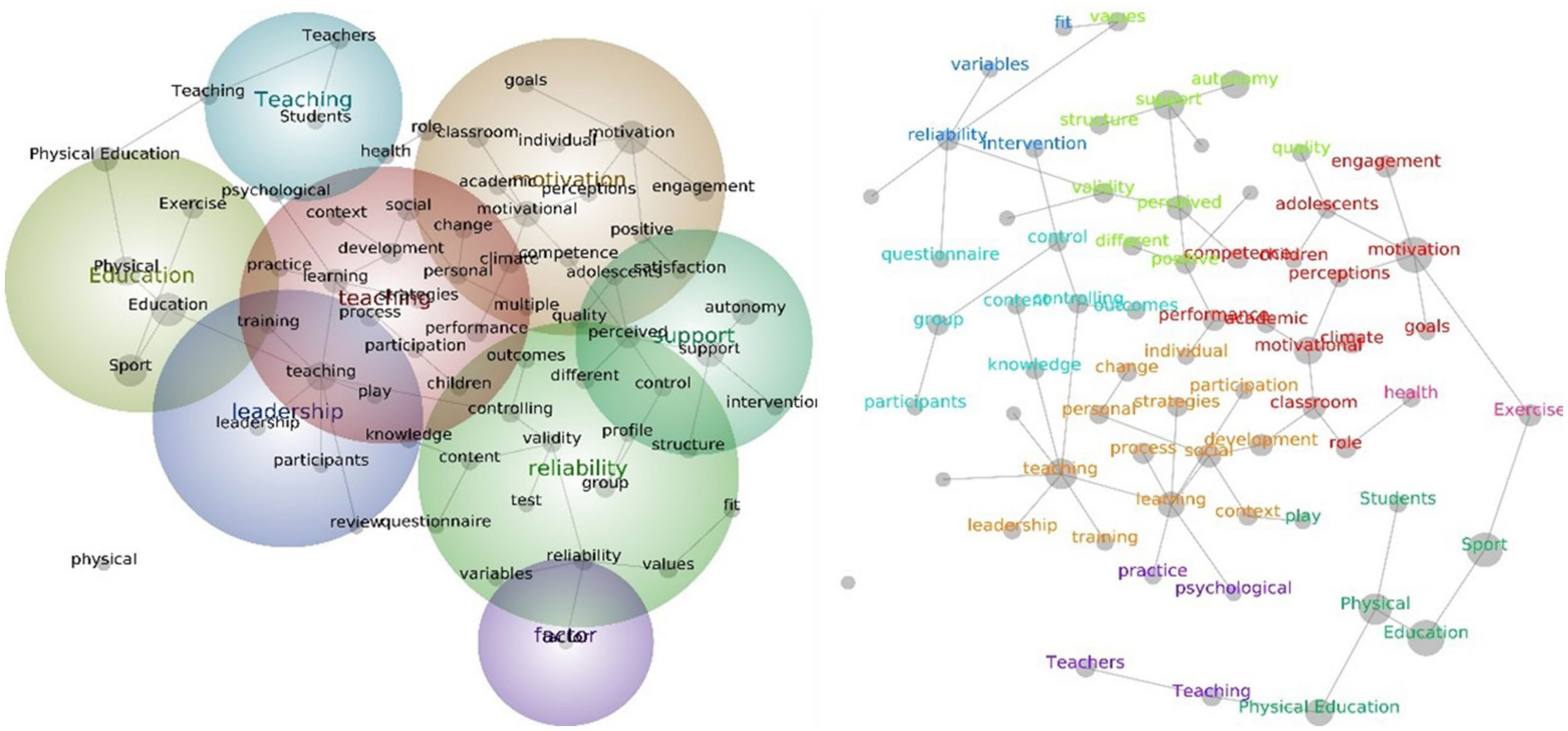
Social network and concept map views for the overall PE teacher leadership studies. The relative size of each theme circle is the boundary of clustered concepts; hot colors (red, orange) indicate the most important themes and cool colors (blue, green) demote those less important.

Overall, eight major themes were confirmed by the data mining analysis conducted in Phase 1 throughout the selected articles (see Table 1). The theme education had the highest significance in terms of hit count and connectedness, followed by motivation, teaching, support, group, engagement, values, and exercise. The education theme contains word concepts such as “sport,” “physical,” and Physical Education” while the motivation theme contains several word concepts including “classroom, ““development, ““performance, ““climate, ““academic, “process,” and “participation.” The support theme contains word concepts such as “autonomy,” “perceived,” “positive,” “outcomes,” “content” and “satisfaction,” while the teaching theme comprises word concepts such as “learning,” “social,” “competence,” “personal” and “multiple” (Figure 3).

**Table 1:** Thematic and conceptual analysis findings resulted from the selected articles by the origins of subjects.

Overall PETL studies	Theme (HC)	Motivator (488)	Education (615)	Support (275)	Teaching (318)
		Group (247)	Engagement (203)	Values (180)	Exercise (383)
		Teaching (176)			
	Concepts (HC/RP)	Motivation (196/66%), teaching (126/43%), support (91/31%), learning (75/25%), engagement (75/25%), autonomy (71/24%), reliability (67/23%), group (67/23%), social (58/20%), values (51/17%), motivational (46/16%), perceived (46/16%), development (45/15%), competence (39/13%), controlling (39/13%), fit (39/13%), validity (37/12%), different (36/12%), knowledge (36/12%), leadership (36/12%), performance (35/12%), classroom (34/11%), intervention (34/11%), climate (32/11%), positive (32/11%)			
ASIAN PETL studies	Theme (HC)	Autonomy (62)	Performance (64)	Teaching (52)	Experiment (52)
		Exercise (80)	Leadership (25)	Autonomy (15)	Group (11)
		Questionnaire (13)	Instruction (3)		
	Concepts (HC/RP)	Autonomy (30/47%), agentic (22/34), support (21/33%), engagement (19/30%), positive (18/28%), leadership (17/27%), performance (16/25%), physical (16/25%), factor (14/22%), motivation (10/16%), teaching (10/16%), fit (10/16%), negative (9/14%), questionnaire (9/14%), belief (8/12%), perceived (7/11%), experimental (7/11%), condition (7/11%), knowledge (7/11%), controlling (6/9%), development (6/9%), control (6/9%), group (6/9%), social (6/9%), change (6/9%)			
North American PETL studies	Theme (HC)	Learning (209)	Climate (132)	Physical Education (151)	Health (4)
	Concepts (HC/RP)	Teaching (50/100%), motivation (40/43%), social (30/33%), development (26/28%), learning (25/27%), interest (25/27%), practice (25/27%), personal (24/26%), climate (22/24%), engagement (15/16%), performance (14/15%), classroom (14/15%), caring (14/15%), support (14/15%), variables (14/15%), children (13/14%), participants (13/14%), play (13/14%), evaluation (12/13%), reliability (12/13%), others (11/12%), relatedness (11/12%), positive (11/12%), different (11/12%), task (10/11%)			
European PETL studies	Theme (HC)	Support (589)	Motivation (338)	Education (348)	Values (146)
		Teachers (149)			
	Concepts (HC/RP)	Motivator (141/97%), physical (97/66%), teaching (87/60%), support (82/56%), autonomy (59/40%), competence (53/36%), group (44/30%), relationship (41/28%), frustration (41/28%), values (41/28%), sport (40/27%), different (38/26%), lesson (38/26%), differences (37/25%), quality (36/25%), skills (36/25%), satisfaction (36/25%), learning (35/24%), engagement (32/22%), controlling (31/21%), intervention (30/21%), life (29/20%), social (29/20%), motivational (27/18%), factor (27/18%)			
Oceania PETL studies	Theme (HC)	Teaching (132)	Motivation(76)	Physical Education (34)	Primary(4)
		Health(9)			
	Concepts (HC/RP)	Motivation (37/88%), teaching (32/76%), learning (32/76%), social (16/38%), knowledge (14/33%), group (14/33%), relatedness (12/29%), work (11/26%), cultural (11/26%), skills (10/24%), support (10/24%), intervention (10/24%), student-centered (9/21%), development (9/21%), critical (9/21%), program (9/21%), health (9/21%), process (8/19%), control (8/19%), content (8/19%), participants (7/17%), controlled (7/17%), role (7/17%), educational (7/17%), curriculum (7/17%)			

PETL, physical education teacher leadership; HC, hit count; RP, relevancy percentage. The relevancy percentage is the percentage frequency of text segments coded with that concept from the origins of the participants sector; all themes analyzed by the Leximancer program are presented in the row, “Theme”; top 25 concepts are listed in the row.

**Figure 4:**
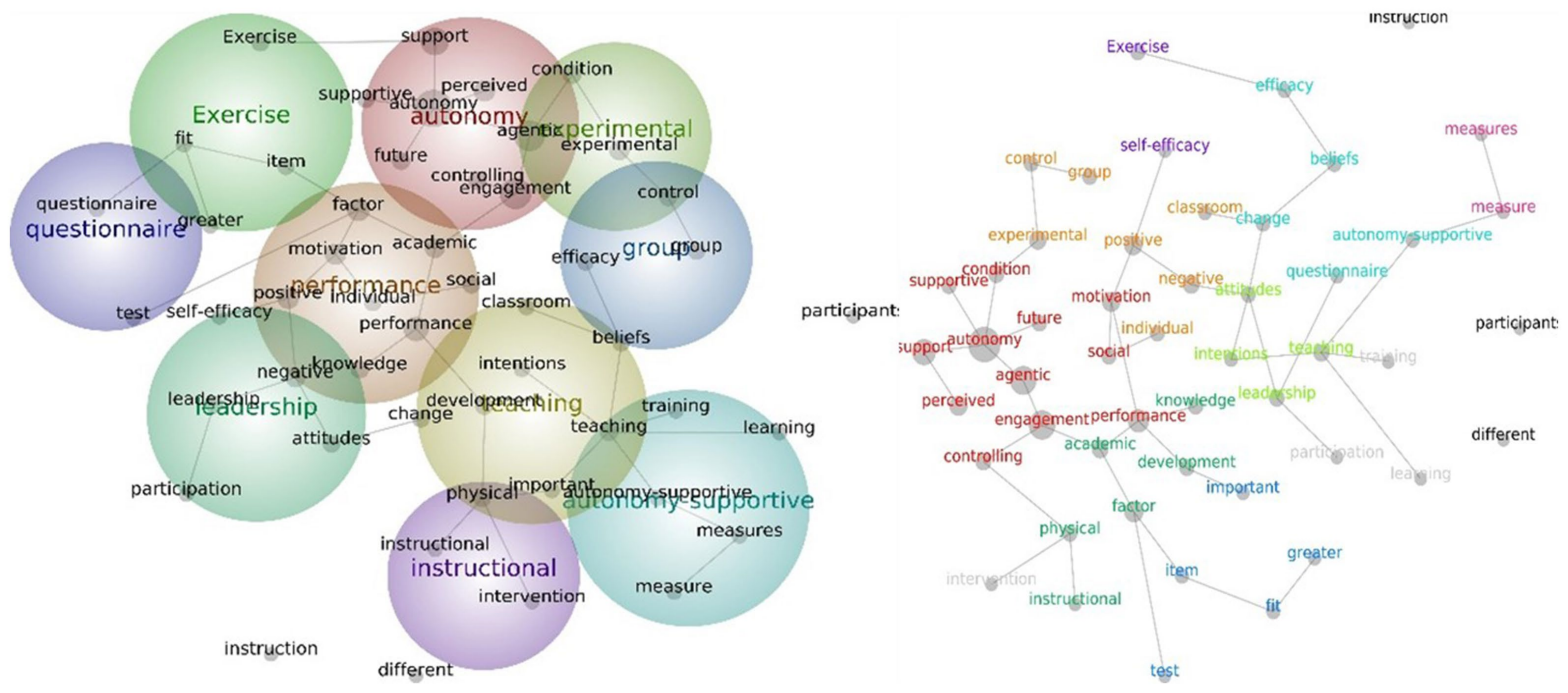
Social network and concept map views for the Asian PE teacher leadership studies. The relative size of each theme circle is the boundary of clustered concepts; hot colors (red, orange) indicate the most important themes and cool colors (blue, green) demote those less important.

For the themes and concepts generated from all studies categorized according to continent, the results showed that, for Asian studies, the most prominent theme that emerged was autonomy, followed by performance, teaching, and experimental. The autonomy theme contains the word concepts such as “engagement,” “agentic,” “support,” “perceived,” “controlling,” and “supportive.” The performance theme includes word concepts such as “motivation,” “positive,” “negative,” “academic,” “knowledge,” and “individual.” The teaching theme contains word concepts such as “development,” “beliefs,” “physical,” “change,” and “intentions,” while the experimental theme has word concepts such as “condition” and “control” (Figure 4).

**Figure 5:**
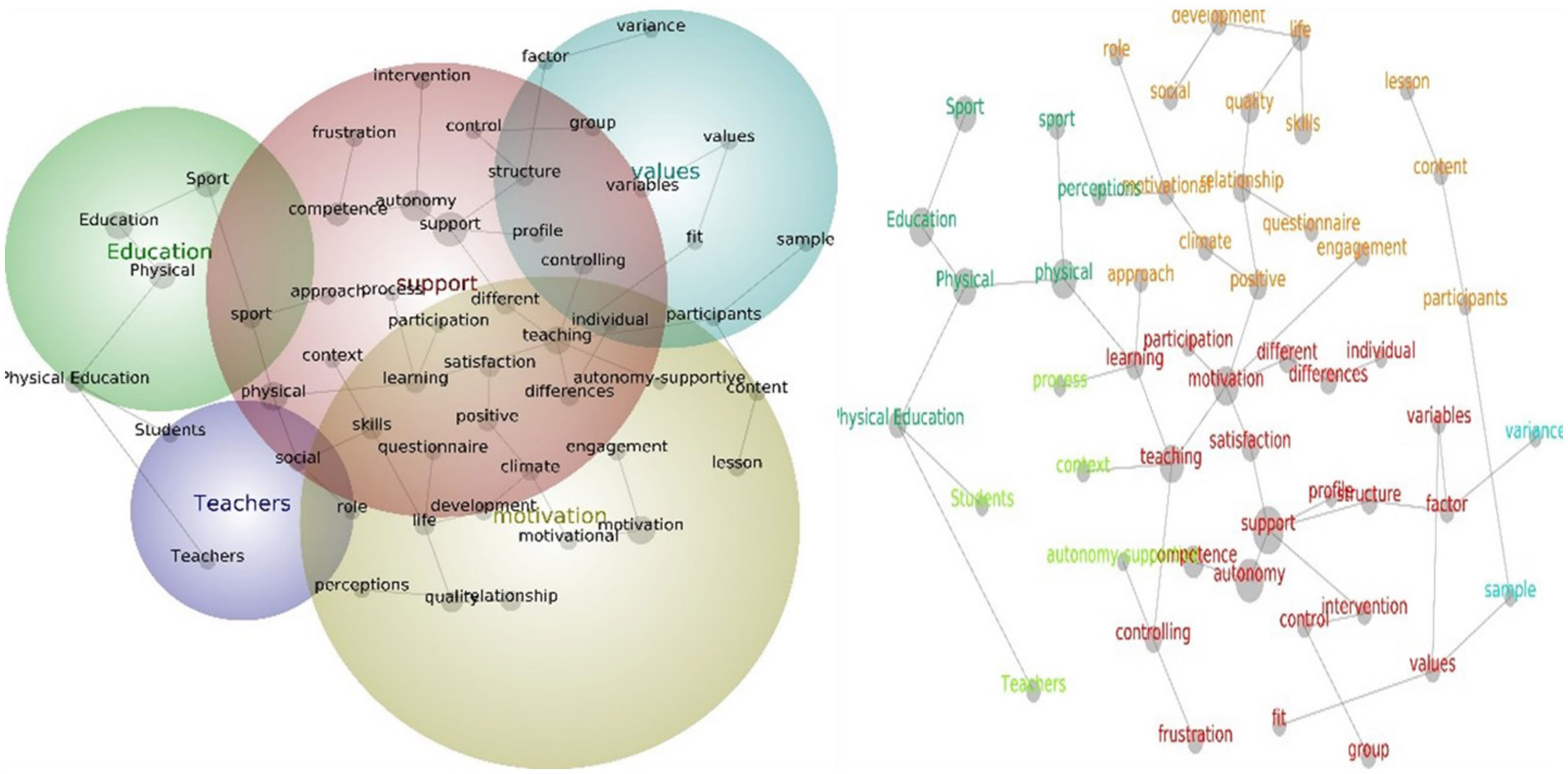
Social network and concept map views for the European PE teacher leadership studies. The relative size of each theme circle is the boundary of clustered concepts; hot colors (red, orange) indicate the most important themes and cool colors (blue, green) demote those less important.

In European studies, only five major themes have been generated by text-mining analysis. These themes are support, motivation, education, values, and teachers themes. The support theme emerged as the highest theme with word concepts such as “autonomy,” “physical,” “teaching,” “competence,” “satisfaction,” positive,” “frustration, and “differences.” The motivation theme contains word concepts such as “quality,” “relationship,” “life,” “engagement,” “climate,” and “autonomy-supportive,” and “questionnaires.” The education theme includes word concepts such as “physical” sport” and “physical education” (Figure 5).

**Figure 6:**
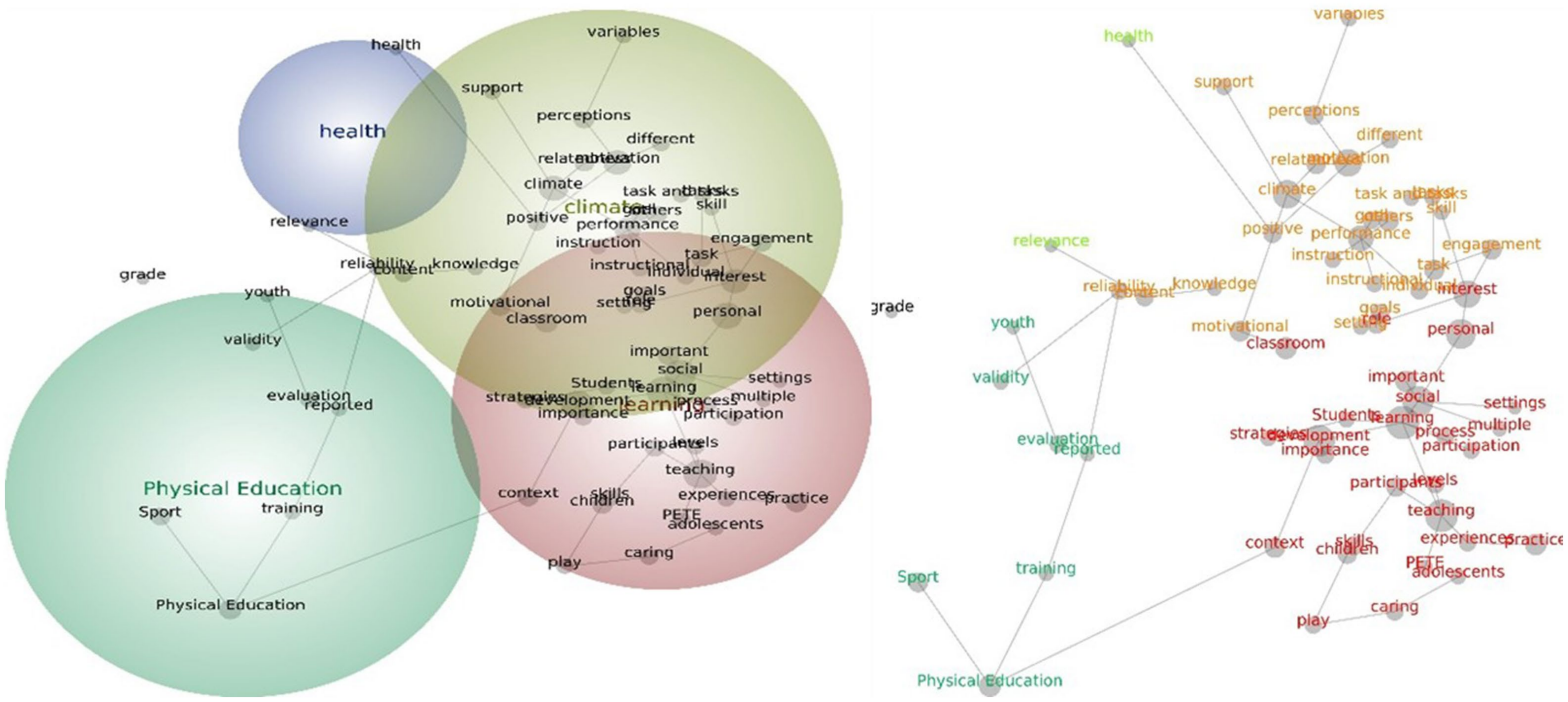
Social network and concept map views for the North American PE teacher leadership studies. The relative size of each theme circle is the boundary of clustered concepts; hot colors (red, orange) indicate the most important themes and cool colors (blue, green) demote those less important.

In North American studies, the most prominent theme is learning, followed by climate, physical education, and health. The learning theme contains word concepts such as “teaching,” “development,” “social,” “classroom,” “interest,” “practice,” “caring,” “role,” and “process.” The climate theme includes word concepts such as “motivation,” “performance,” “engagement,” “relatedness,” “goals,” “perceptions,” and “different.” The physical education theme only contains the word concept “sport” whereas the health theme includes only has its own word concept (Figure 6).

**Figure 7:**
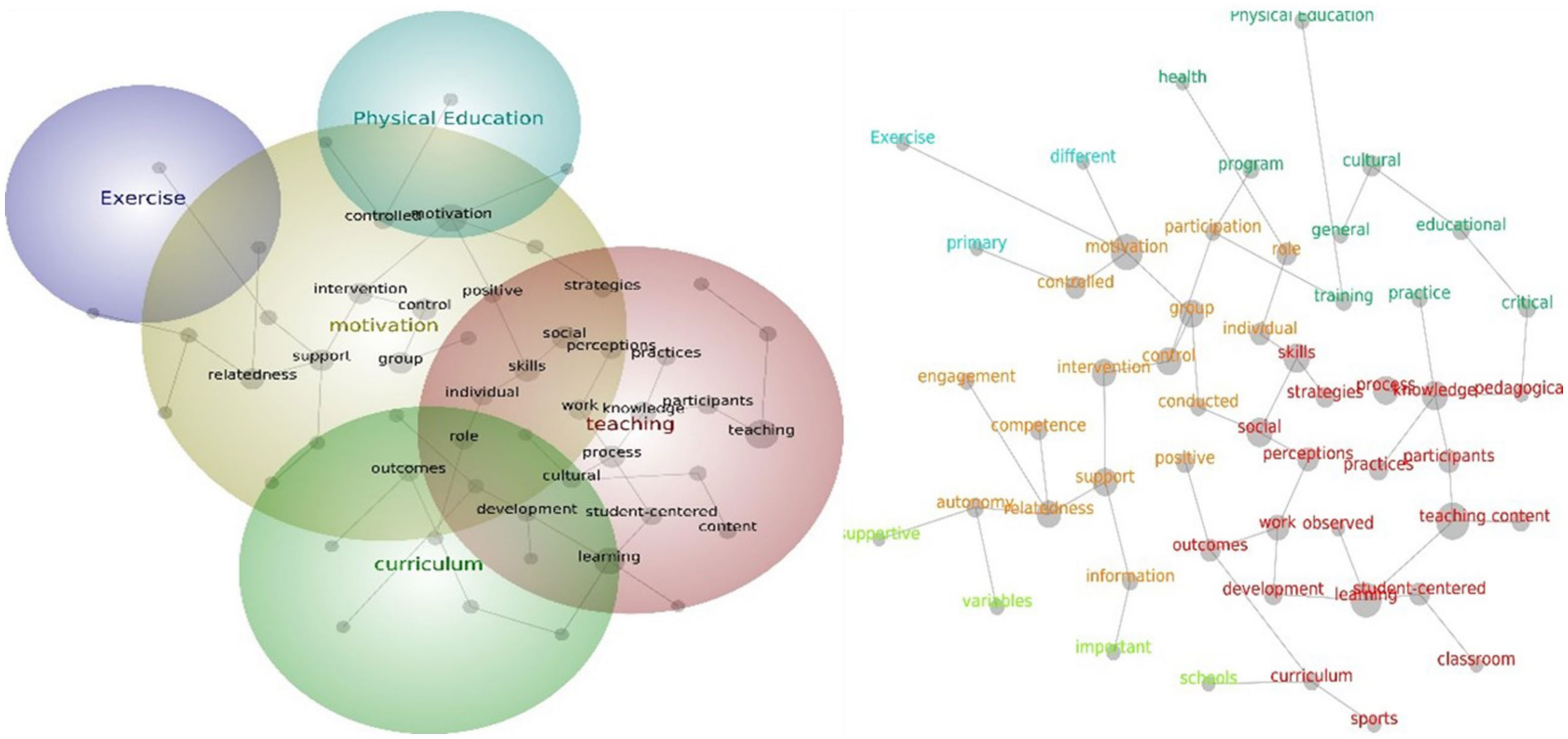
Social network and concept map views for the Oceania and others PE teacher leadership studies. The relative size of each theme circle is the boundary of clustered concepts; hot colors (red, orange) indicate the most important themes and cool colors (blue, green) demote those less important.

Five themes were assessed as being relevant to Oceania and other studies. The theme that emerged was teaching, followed by motivation, physical education, primary education and health. The teaching theme includes word concepts such as “learning,” “knowledge,” “skills,” “social,” “process,” “participants,” “student-centered,” “development,” and “strategies.” The motivation theme contains word concepts such as “control,” “group,” “support,” “interventions,” “positive,” and “autonomy.” The remaining three themes only included their own word concepts (Figure 7).

### 3.3 Findings from the meta-analysis

This study incorporated the following student-related outcome variables: (1) satisfaction; (2) amotivation; (3) engagement; (4) motivation; (5) frustration; and (6) competence. When examining the themes and word concepts generated from Phase 1, as well as the common dependent variables measured in the included quantitative studies related to PE teacher leadership, these outcome variables have consistently held considerable importance (Table 2).

Regardless of gender, the meta-analysis revealed that the relationship between the autonomy-supportive behavior of physical education instructors and student satisfaction had moderate to substantial effects (ES ranged from.456 to.582; 95% CIs =0.461, 0.572; *k* = 59; *p* < 0.05). Based on student sex, male students exhibited a greater degree of satisfaction (ES: male = 0.582; 95% CIs = 0.538, 0.622; *k* = 28; *p* < 0.05) compared to female students.

The relationship between student amotivation and supportive behaviors of physical education teachers exhibited negative effect sizes, with values ranging from −0.00788 to −0.252 (95% CIs = −0.239, −0.034; *k* = 15; *p* < 0.05). Based on student sex, the ES value for the relationship was more negative for the female student group (ES female = −0.254; 95% CIs = −0.123, −0.377; *k* = 8; *p* < 0.05).

The effect sizes for the correlation between supportive behaviors of physical education instructors and student engagement were predominantly positive (ES ranged from.346 to.400; 95% Cis =0.239, 0.453; *k* = 23; *p* < 0.05). Based on student sex, male students exhibited a greater degree of engagement (ES male = 0.400; 95% CIs = 0.360, 0.438; *k* = 2; *p* < 0.05) compared to female students.

The effect sizes ranging from.434 to.522 (95% CIs =0.404, 0.541; *k* = 24; *p* < 0.05) for the association between PE teachers’ supportive behavior and student motivation were also within the positive range. Based on student sex, this relationship was associated with a relatively higher ES value among female students (ES female = 0.522; 95% CIs = 0.428–0.606; *k* = 11; *p* < 0.05).

The relationship between supportive behaviors of physical education teachers and student frustration exhibited negative effect sizes, with values ranging from −0.00712 to −0.205 (95% CIs = −0.190, 0.005; *k* = 15; *p* < 0.05). Based on student sex, the ES value for the relationship was more negative for the female students (ES female = −0.00712; 95% CIs = −0.190, 0.005; *k* = 8; *p* < 0.05).

The effect sizes ranging from.198 to.440 (95% CIs =0.171, 0.459; *k* = 14; *p* < 0.05) for the relationship between the supportive behavior of PE instructors and student competence were moderate. Based on student sex, male students (ES = 0.440) exhibited a greater degree of competence compared to female students (ES = 0.198).

**Table 2:** Effects of autonomous support of PE teachers’ behavior on student outcome variables.

Variables	Gender Segmentation	*k*	ES	−95%CI	+95%CI	*Q*	*I* ^2^	Tau
Satisfaction	Overall	59	0.518	0.461	0.572	4039.327	98.564	8.63E-02
	Female	31	0.456	0.345	0.555	3184.715	99.058	0.138782
	Male	28	0.582	0.538	0.622	643.107	95.801	2.78E-02
Amotivation	Overall	15	−0.138	−0.239	−0.034	567.562	97.533	4.14E-02
	Female	8	−0.254	−0.377	−0.123	172.094	95.932	3.66E-02
	Male	7	7.88E-03	−1.26E-02	2.83E-02	167.657	96.421	0.020951
Engagement	Overall	23	0.351	0.239	0.453	2257.753	99.025	8.79E-02
	Female	21	0.346	0.220	0.461	2204.881	99.092	0.101616
	Male	2	0.400	0.360	0.438	2.269	55.946	6.35E-04
Motivation	Overall	24	0.476	0.404	0.541	983.194	97.660	4.69E-02
	Female	11	0.522	0.428	0.606	297.234	96.635	4.07E-02
	Male	13	0.434	0.324	0.532	679.525	98.234	0.05441
Frustration	Overall	10	−9.77E-02	−0.201	8.54E-03	237.890	96.216	2.83E-02
	Female	8	−7.12E-02	−0.190	5.00E-02	200.468	96.508	0.029497
	Male	2	−0.205	−0.356	−4.53E-02	8.284	87.929	1.23E-02
Competence	Overall	14	0.323	0.171	0.459	998.725	98.698	9.28E-02
	Female	7	0.198	−4.17E-02	0.416	465.499	98.711	0.10464
	Male	7	0.440	0.322	0.544	174.342	96.558	3.29E-02

*k, number of comparisons; CI, confidence interval; Q, Q-value; I2, I-Squared; Tau, Tau-Squared.*

## 4 Discussion

This study systematically synthesized and evaluated pertinent qualitative and quantitative literature on PE teachers’ leadership styles and behaviors and their associations with student outcomes using a mixed methods (HDST) approach to examine both lexical and statistical data through data mining and meta-analyses, respectively.

Based on the findings in the first phase of the HDST technique, we found that overall, eight (8) themes emerged to be associated with PE teacher leadership. Motivation, education, support and teaching themes were identified as major themes with connectivity scores of 931,593,511, and 461, respectively, indicating their relative importance. Primary concepts contained within these major themes include: “classroom,” “development,” “performance,” “climate,” “process,” “strategies,” “adolescents,” “academic,” “play” and “participation” under the motivation theme; “sport” and “physical education” under the education theme; “autonomy,” “perceived,” “positive,” “outcomes,” “satisfaction” under support theme; and “learning,” “social,” “competence,” “personal,” and “training” under teaching theme. These major themes together with their connected concepts indicate the principal roles of PE teachers in educating students about physical education and achieving student outcomes by providing a motivational classroom climate, as well as offering quality teaching, autonomy, and support to individuals or groups of students, leading to positive outcomes such as motivation, competence, satisfaction, and learning in PE. This finding substantiates the idea that PE teachers are important educational leaders who can facilitate motivation and other PE-related outcomes of students by demonstrating positive leadership styles and teaching behaviors, creating fun and meaningful PE class settings, and providing additional support for the transformational leadership approach. In addition, the relevant themes and concepts connected with these themes are in line with the Self-Determination Theory (SDT) (Deci and Ryan, 2012) which proposes how social factors can either enhance or undermine an individual’s volitional form of motivation, well-being, and performance. Particularly, the level of intrinsic motivation that can be influenced by a person’s perceived support or restraint of their psychological needs (i.e., competence and autonomy) in a social context (PE class). This theoretical approach is evident in most of the articles included in this study (Cheon et al., 2022; Liu and Chung, 2017; Reeve et al., 2020) which resulted in a conceptual map with word concepts that are highly relevant and interlinked with this theoretical framework. The finding provides evidence that these theoretical approaches have concepts that can shed light in understanding human behaviors, especially how situational or external variables such as a leader (i.e., teacher) can impact responses of followers (students).

The minor themes that were generated, which include engagement and values contained word concepts such as “structure,” “questionnaire,” “variables,” “reliability,” “validity,” and “children,” were found to be semantically connected, suggesting the type of research methodology conducted in this topic and the study participants from whom they collected the responses. This finding is not surprising, given the large number of articles that employed quantitative methods to examine PE teacher leadership, accounting for more than 70% of the included studies. However, it is interesting to point out that, through the use of the data mining tool, this quantitative information about the general profile of studies was also effectively identified via the connectivity of relevant themes and concepts generated from the automatic and systematic extraction, inspection, and analysis of each text contained in the entire set of articles. Moreover, with the available conceptual map visually displaying a network of interconnected concepts, detecting and classifying important themes and their relationships is much faster and more efficient, demonstrating that statistical records can also be reflected through textual outputs via data mining technology.

### 4.1 Concepts and themes based on regions (Asia, Europe, North America, Oceania and others)

Comparing the relevant concepts and themes based on the regional origins of published articles, the results showed that while the number of significant themes varied among regions (Asia: nine; Europe: five; North America: four; Oceania and others: four), each region’s key concepts within the major themes were very similar, showing strong relationships with PE teacher leadership behaviors and student outcomes. For instance, high level concepts such as “student-centered,” “social process,” “caring,” “development,” “support,” “autonomy,” “learning,” and “teaching” reflect the PE teacher’s teaching process and behaviors to carry out with students as well as the kind of classroom environment to cultivate during PE class. Other relevant concepts that are similar among regions include “motivation,” “performance,” “perceptions,” “positive,” negative” “knowledge, “and “engagement, “indicating the student outcomes commonly examined by researchers to be influenced by PE teacher leadership.

Interestingly, the word “health” was not generated as a relevant concept in Asia and Europe but was represented in North America, Oceania, and others. This discrepancy indicates that studies from the latter regions have managed to highlight the concept of health as an important aspect of PE and the significant role of PE teachers’ leadership in facilitating the promotion of health. However, this emphasis on health is somewhat less pronounced in studies from Asia and Europe, which are estimated by the text-mining tool as an insignificant concept. Perhaps the literature included within these regions still places considerable emphasis on PE curriculum content, which is predominantly toward motor and sports skill development, performance-or competition-based programs, and physical activity participation (Katsarova, 2016; Hardman, 2008). In turn, PE teachers may feel obligated to prioritize these aspects in their teaching, which eventually led researchers to investigate the effectiveness of PE teachers’ leadership and teaching behaviors in delivering PE lessons, using traditional class-related outcome assessment tools to gauge students’ perceptions and behaviors regarding their PE learning experiences.

Looking at the significant themes and highly relevant concepts generated in Asian and European studies, it is apparent that the connectivity of concepts mainly illustrates the direct effects of PE teachers’ supportive and controlling teaching behaviors on certain behavioral (e.g., performance and engagement) and psychological (e.g., motivation) components of student learning in PE settings. Based on these findings, despite the fact that PE curricular reforms have been undertaken in recent years, incorporating the promotion of lifelong active and healthy living (D'Anna et al., 2019; Ha et al., 2008; Lee and Cho, 2014) among students, knowledge is still scarce when it comes to examining students’ perceptions of their PE teachers’ behaviors and instructions in cultivating the value and development of health and well-being as a learning aspect of PE. Therefore, more studies are needed to examine PE teachers’ leadership in achieving the holistic goals of PE among students.

### 4.2 Relationship between PE teacher leadership and student outcomes

Overall, the results from Phase 2 of the HDST revealed small to large effects of PE teachers’ transformational behaviors on students’ learning outcomes. Particularly, PE teacher autonomy support had a strong relationship with student satisfaction and a moderate relationship with student motivation, engagement, and competence. By contrast, student amotivation and frustration had negative but very small associations with PE teacher autonomy support. This indicates that when students perceive that their PE teachers display leadership behaviors that support their autonomy, they are likely to experience greater levels of satisfaction and moderate levels of competence and engagement in PE. Moreover, students’ feelings of amotivation and frustration tend to decrease with perceived autonomy support from their PE teachers. The present findings are consistent with previous meta-analyses that examined similar outcome variables related to PE teachers’ behaviors (Lochbaum and Jean-Noel, 2016; Tao et al., 2022; Vasconcellos et al., 2020) which also showed comparable effect sizes. Additionally, the results align with the transformational teaching framework, wherein transformational teaching behaviors are assumed to affect students’ intrapersonal psychological variables such as intrinsic motivation, self-efficacy, and attitudes, which then lead to behavior change. In this context, the transformational behaviors demonstrated by the PE teacher that nurture autonomy of students such as trusting them to make their own decisions, stimulating their critical thinking and other high-level cognitive skills, and empowering them to speak their minds, corresponds to idealized influence, intellectual stimulation, and inspirational motivation, respectively. When PE teachers utilize these behaviors, it can improve students’ attitudes toward the teacher, increase their motivation in PE, enhance satisfaction and self-confidence, and reduce feelings of amotivation and frustration and eventually lead to greater student engagement in PE.

This result provides additional evidence about the importance of PE teachers’ positive teaching and leadership, specifically behaviors that cultivate student autonomy, in developing various learning outcomes in students during PE classes. This finding also underscores that the influence of PE teachers’ leadership may vary in degree depending on the type of learning domain being evaluated.

### 4.3 Relationship between PE teacher leadership and student outcomes based on gender

When examining gender as a moderating variable, the results showed that the effect of PE teacher leadership on student satisfaction, competence, and engagement was higher for males (satisfaction ES = 0.582; competence ES = 0.44; engagement ES = 0.400) than for females (satisfaction ES = 0.456; competence ES = 0.198; engagement ES = 0.346), while student motivation was greater for females (ES = 0.522) than for males (ES = 0.434). These findings suggest that perceptions of satisfaction, competence, and engagement tend to be higher in males than in females, and motivation tends to be higher in females than in males when PE teachers display high levels of positive and autonomy-enhancing leadership behaviors.

The differences in perceptions [i.e., satisfaction, competence, engagement, (a)motivation, and frustration] between male and female students are likely to be associated with the gender of the PE teacher, the degree and type of interactions the PE teacher shows the students, and the students’ preferences for certain PE behaviors. Nicaise et al. (2007) examined PE teacher-student interactions during PE classes and reported that PE teachers gave more frequent praise, technical information, encouragement, criticism, organization, and misbehavioral feedback to male students, whereas they provided more positive reinforcements such as technical information and encouragement to girls. In another study, female PE teachers were observed to have fewer interactions with female students than male students, whereas male PE teachers had fewer interactions with male students than with female students (Davis and Nicaise, 2011). Finally, in a similar study on sports, the leadership preferences of young male and female athletes differed based on the gender of the coach. In particular, boys preferred that their male coaches display fewer occasions of democratic, autocratic, and social support than female coaches. Conversely, girls preferred that their male coaches show more frequent democratic, autocratic, and social support leadership behaviors than female coaches. Based on these previous findings, when male and female students perceive that their male and female PE teachers demonstrate positive leadership behaviors, such as supporting students to become independent learners and motivating them to explore innovative ways to play games and engage in physical activities during class, they are likely to feel greater satisfaction, motivation, engagement, and competence during PE class and experience slightly lower levels of frustration and amotivation.

In general, the present results highlight not only the importance of student gender as a moderating variable in the relationship between PE teacher leadership and student outcomes but also showcase the dynamics among the personal characteristics of both students and PE teachers. More importantly, this study adds new knowledge to the literature on PE leadership, addressing the call from previous researchers to examine this factor (Guo et al., 2023).

### 4.4 Divergence and convergence between qualitative and quantitative findings

Based on the overall summary from examining all textual documents using the automated text analysis, the words “motivation” and “satisfaction” are found to be relevant word concepts with high connectivity with PE teachers. Furthermore, these two concepts acquired the highest ES values among the outcome variables examined in the meta-analysis. The correspondence in the results between the semantic and statistical information underscores the important function of the emergent themes and concepts from processing qualitative data in guiding researchers regarding which variables are worth exploring as potential outcome variables to undergo meta-analysis.

On the other hand, based on the overall result from the qualitative data, “health” was identified as a relevant concept with distinct connections with “student” and “goals” concepts contained in the teaching theme. This indicates that teaching PE is vital for students to achieve their health goals. Based on the text mining results, the teaching theme’s connectivity score (129), compared with the main themes motivation (931), education (593), and support (511) was considerably low, suggesting very weak associations with these important themes and concepts. However, of all the included quantitative articles, no researchers directly examined whether students explicitly recognized the value of PE for health improvement through the teaching behaviors of their PE teachers, thereby reflecting a discrepancy between the qualitative and quantitative outcomes.

In many countries, the PE curricula have been revised to meet learners’ current needs. From PE objectives mainly focused on developing students’ motor and sport skills, current PE programs recently incorporate objectives that emphasize fostering the promotion of healthy lifestyle and the value of physical activity for fitness, and health (D'Anna et al., 2019; Kurokawa et al., 2020; SHAPE America, 2024). To determine whether the additional health-related objectives of PE education are truly effective, it is just appropriate the learning outcomes should be evaluated based on empirical evidence.

However, the present study’s quantitative and qualitative findings reveal that a knowledge gap still exists regarding the influence of PE teachers’ leadership on students’ thoughts, attitudes, and behaviors toward PE and physical activities, above and beyond the frequently evaluated student-related outcomes (i.e., satisfaction, enjoyment, and motivation). Therefore, it is suggested that more comprehensive research be conducted on the role of PE teacher leadership. In particular, the scope of future studies should be expanded by examining outcome variables focused on students’ experiences of their PE teachers’ behaviors in communicating the value of PE and physical activity in developing and optimizing their overall health. Examining this topic not only sheds light on how PE teachers’ leadership influences students’ active participation in PE and subsequently resonates with students’ lifelong involvement in physical activities but also contributes to the PE leadership literature.

The meta-analysis conducted as part of this review allowed us to investigate student gender as a moderating variable in the relationship between PE teacher leadership and student-related outcomes. From the results, we found that the magnitude of the PE teacher leadership-student outcome relationships differed between male and female students. While moderating variables are commonly examined in meta-analysis when there is substantial heterogeneity in the included studies, interestingly, the word “gender” or “sex” was not found as relevant concept in the results of the qualitative analysis. This corroborates the findings of a previous leadership review that employed the HDST mixed-method approach (Kim and Cruz, 2022). Therefore, the findings highlight that when examining textual documents using content analysis, the absence of relevant concepts pertaining to the personal characteristics of study participants can offer valuable information that may guide researchers in identifying potential variables that can be further explored quantitatively, which in turn facilitates the understanding of a particular research topic through both macro-and micro-level approaches.

### 4.5 Implications

Following the findings of the present study, for researchers investigating the leadership phenomenon in PE settings, it is recommended that future studies focus more on evaluating students’ cognition of the values they learn every time they attend PE classes, as well as how PE teachers overtly communicate these positive values to students. This involves using or creating evaluation tools in the form of questionnaires that measure students’ appraisals toward the meaning of PE and physical activities for health attainment, enhancement, and maintenance. Putting additional emphasis on evaluating the cognitive aspects of students’ perceptions of their (non)participation in PE through the behaviors of their PE teachers would provide important information for a better understanding of the complex dynamics of PE teachers’ leadership and its impact on student outcomes.

For PE teachers, it is recommended that they should be aware of their students’ responses when displaying certain behaviors during PE class. Given the findings that PE instructors’ autonomy supportive behaviors have positive (satisfaction, motivation, competence, engagement) and negative (amotivation and frustration) relationships with student outcomes, PE teachers should strive to frequently demonstrate transformational leadership behaviors which also correspond with autonomy-supportive actions. One strategy is for PE teachers to display intellectual stimulation transformational behaviors focused on encouraging students’ autonomy. This can be achieved by stimulating students’ decision-making and problem-solving abilities. For instance, PE teachers could ask students to choose which physical activities would best suit their individual physical fitness level and motor skill capabilities. By giving students the opportunity to experience analyzing and evaluating options independently, rather than dictating them, they are likely to show positive emotions, attitudes, and behaviors toward the teacher and the class. In addition, considering the present findings that PE teacher leadership behaviors can affect male and female students’ outcomes differently, developing new teaching strategies or adopting certain behaviors that would cater to the preferences of each group of students may eventually lead to a more motivational classroom climate and, in turn, better PE engagement. Previous studies have shown that male and female students have different preferences in PE classes, with female students favoring individual sports for fun, while male students enjoying team sports to exercise and build fitness (Cruz, 2021; Kuśnierz et al., 2020). In this case, to empower students and give them a voice in choosing activities during PE sessions, teachers can exhibit autonomy-supportive transformational behaviors such as inspirational motivation and individualized consideration. These behaviors include tailoring instructions and providing specific, constructive feedback (individualized consideration), as well as offering words of encouragement to promote greater effort and engagement in PE and communicating clear expectations about student behavior, health progress, and performance (inspirational motivation).

School administrators can invest in professional development programs that enhance PE teachers’ transformational leadership behaviors and teaching skills. These programs should focus on understanding the principles of transformational leadership and their application to both educational settings in general and physical education (PE) in particular (Beauchamp and Morton, 2011). Additionally, hands-on workshops can provide practical guidance on how, when, and where to demonstrate transformational behaviors in PE classes, as well as which students to focus on. By participating in such training, PE teachers will be better equipped to apply effective strategies based on transformational leadership principles, optimizing student experiences in the classroom while avoiding teaching behaviors that might elicit negative responses from students.

### 4.6 Limitations

The present study has limitations that need disclosure. First, only six student outcomes (satisfaction, motivation, engagement, competence, frustration and amotivation) were examined in the meta-analysis part of this study. However, these dependent variables were selected using automated processing, substantially mitigating researcher bias while increasing the identification of meaningful variables to evaluate, thereby facilitating researchers’ decision-making in terms of the research variables worthy of exploration. Nonetheless, compared with previous reviews, the number of outcome variables examined related to student perceptions about their PE teachers’ behaviors is similar to Lochbaum and Jean-Noel (2016), more than Guo et al., (2023) and Tao et al. (2022), but less than Vasconcellos et al. (2020), and therefore provides additional insights about the impact of PE teacher leadership on student outcomes.

Second, only gender was evaluated as a moderating variable in the relationship between PE teacher leadership and student outcomes. While we rigorously screened all quantitative studies to identify other potential moderators for this relationship (e.g., student age or academic level, PE teacher’s gender), due to the lack of statistical data from the quantitative studies, we were unable to explore these variables. Hence, additional studies are suggested on this topic so that future researchers reviewing studies related to PE teacher leadership can precisely identify factors that may significantly affect students’ positive and negative perceptions toward PE and physical activities.

Third, the analyses only included published articles with accessible full-texts, so some relevant studies on this topic may not have been included in the review. However, we made every effort to reach out to the authors for copies of their papers if the full texts were not available through our library repository. Likewise, another limitation of the study is that some articles were excluded due to a lack of agreement among authors. It is unknown how these articles might have contributed to our understanding of the topic.

Finally, the leadership behavior of PE teachers examined in the meta-analysis was limited to autonomy support, as this was explicitly highlighted in the overall results of the content analysis. This finding may be attributed to the fact that most of the included studies were grounded in Self-Determination Theory (SDT) when investigating the influence of PE teachers on student outcomes. Therefore, researchers are encouraged to further explore this topic through the lens of the transformational leadership framework, as transformational behaviors displayed by teachers and leaders have been shown to predict adaptive cognitive, emotional, and behavioral responses in students and followers (Beauchamp and Morton, 2011). By using this paradigm, we can better understand the relationship between the transformational leadership behaviors of PE teachers in general, and their specific four dimensions in particular, on various student outcomes.

## 5 Conclusion

Overall, by reviewing both qualitative and quantitative studies on PE teacher leadership, we found that the autonomy-supportive and controlling leadership behaviors of PE teachers are important themes that are strongly connected with the classroom motivational climate and students’ positive perceptions of PE, particularly motivation, satisfaction, competence, and engagement. Second, noticeable variances were found in the major themes and relevant word concepts generated among the included studies, based on the main author’s regional affiliation. Third, the concept of health is not a prominent topic explicitly evaluated in relation to leadership in the PE domain. Fourth, PE teacher autonomy support had medium to large positive effects on student satisfaction, motivation, competence, and engagement. PE teachers’ autonomy-supportive leadership had a marginally positive effect on students’ feelings of amotivation and frustration in PE. Fifth, the effect of PE teachers’ autonomy-supportive leadership on students’ perceptions of satisfaction, motivation, engagement, competence, frustration, and amotivation differed between male and female students. Finally, the application of the HDST approach to summarize both qualitative and quantitative articles facilitated an understanding of the topic of leadership in the domain of PE in a more robust and comprehensive manner.

## Data Availability

The original contributions presented in the study are included in the article/Supplementary material, further inquiries can be directed to the corresponding author.
