# Supplementary material for: Leadership practices of physical education teachers and student-related outcomes: a systematic mixed method review and analysis
Source: Front Psychol. 2024 Nov 1;15:1442014. doi: 10.3389/fpsyg.2024.1442014 (PMC11563983; doi:10.3389/fpsyg.2024.1442014)
Supplement: Supplementary file 1 [file Table_1.docx]

Supplementary Table. *List of qualitative and quantitative studies included in the review and meta-analysis using HDST approach*

|  | Authors | Year | Total authors | Country | Type of research | Student  Participants | Leadership/Teaching styles and behaviors assessed | Student outcome  measured |
| --- | --- | --- | --- | --- | --- | --- | --- | --- |
| 1 | Cheon et al. | 2012 | 3 | Korea | Mixed method | Secondary | PE teachers' autonomy-supportive and controlling teaching behaviors | Needs satisfaction in PE - autonomy, competence, relatedness |
| 2 | Hsu & Pan | 2019 | 2 | Taiwan | Mixed method | Secondary | Perceptions of teacher RISE supporting behaviors in PE | Responsibility in PE - effort, self-direction, following class rules, respect for others, helping others, and cooperation |
| 3 | Sato et al. | 2022 | 4 | Japan | Qualitative | Tertiary | Preservice Adapted PE teachers perceptions of teaching | NA |
| 4 | Yang & Dong | 2017 | 2 | China | Quantitative | Tertiary | Leadership style of PE college teachers | Intrinsic and extrinsic learning motivation |
| 5 | Reeve et al. | 2020 | 3 | Korea | Quantitative | Secondary | PE teacher autonomy support | Autonomy satisfaction in PE |
| 6 | Li et al. | 2019 | 3 | Singapore | Quantitative | Tertiary | PE primary and secondary teachers attitudes about integrating STEM education in PE | NA |
| 7 | Behzadnia | 2021 | 1 | Iran | Quantitative | Tertiary | PE teacher' need-supportive and need-thwarting teaching behaviors | Psychological needs satisfaction |
| 8 | Kwon et al | 2010 | 3 | Korea | Quantitative | Secondary | PE teacher-coach perceived leadership behaviors | NA |
| 9 | Liu & Chung | 2017 | 2 | Hongkong China | Quantitative | Secondary | PE teachers Need-supportive teaching style | Psychological Needs Satisfaction in PE |
| 10 | Cheon et al. | 2022 | 4 | Korea | Quantitative | Secondary | PE teachers autonomy-supportive and controlling instructional behaviors | Needs satisfaction in PE - autonomy, competence relatedness |
| 11 | Cheon et al. | 2018 | 3 | Korea | Quantitative | Secondary | PE teachers motivating styles | Satisfaction and frustration in PE |
| 12 | Cheon & Reeve | 2013 | 2 | Korea | Quantitative | Secondary | PE Teachers teaching behaviors | Motivation, amotivation, engagement, skill development, future intention to do PA, PE-specific achievement |
| 13 | Liu et al. | 2018 | 3 | Taiwan | Quantitative | NA | PE teachers self reported perception towards Adapted PE | NA |
| 14 | Tsai | 2017 | 1 | Macau | Quantitative | Tertiary | PE teachers teaching style | NA |
| 15 | Behzadnia et al. | 2018 | 4 | Iran | Quantitative | Tertiary | Perceived PE collge teachers teaching styles and behaviors-autonomy suppotive and controlling styles | NA |
| 16 | Hosseini et al. | 2020 | 3 | Iran | Quantitative | Secondary | Perceived PE teacher autonomy support | Needs satisfaction in PE - autonomy, competence relatedness |
| 17 | Cheon et al. | 2019 | 3 | Korea | Quantitative | Secondary | perceived PE teachers autonomy support | Needs satisfaction in PE - autonomy, competence relatedness |
| 18 | Coral Lim & Wang | 2009 | 2 | Singapore | Quantitative | Secondary | perceived PE teachers autonomy support behaviors | motivation for PE |
| 19 | Cheon et al. | 2019 | 3 | Korea | Quantitative | Secondary | perceived PE teachers Autonomy supportive and controlling teaching | Satisfaction and frustration in PE |
| 20 | Reeve & Cheon | 2016 | 2 | Korea | Quantitative | Secondary | perceived PE teachers autonomy-supportive motivating style | NA |
| 21 | Behzadnia et al. | 2022 | 3 | Iran | Quantitative | Secondary | perceived PE teachers interpersonal behaviors | Satisfaction and frustration in PE |
| 22 | Zhou et al. | 2021 | 4 | China | Quantitative | Elementary | perceived PE teachers OBSERVED teaching behavior | Active participation in PE-MVPA using accelerometer |
| 23 | Hsu et al. | 2022 | 4 | Taiwan | Quantitative | Secondary | Perceived PE teachers RISE Support - positive recognition, task assignment, and relationship establishment | self-efficacy in PE |
| 24 | Choi et al. | 2020 | 7 | Hongkong | Quantitative | Tertiary | Preservice PE teachers | NA |
| 25 | Li et al. | 2021 | 5 | China | Quantitative | Tertiary | University PE teachers perceived teaching behaviors | Behavioral engagement in PE |
| 26 | Sturm et al. | 2021 | 5 | Germany | Mixed method | Secondary | PE teachers' teaching behaviors | PE students physical activity behavior using accelerometer |
| 27 | Haerens et al. | 2013 | 6 | Belgium | Mixed method | Secondary | PE teaching need-supportive behaviors | need support in PE |
| 28 | Lamb et al. | 2021 | 4 | UK | Mixed method | Secondary | perceived PE teachers teaching behaviors | PE students experiences in PE |
| 29 | Manzano-Sanchez et al. | 2020 | 3 | Spain | Mixed method | Secondary | perceived PE teachers' behaviors of promoting values | Academic performance - grade obtained in PE |
| 30 | Girard et al. | 2021 | 3 | France | Mixed method | Secondary | perceived PE teachers social support (emotional, informational, appraisal, and intrumental support) | PE student engagement - based on interview answers |
| 31 | Jung & Choi | 2016 | 2 | UK | Qualitative | Secondary | PE teachers' indirect teaching behaviors | social and moral development |
| 32 | Moen et al. | 2020 | 8 | Norway | Qualitative | NA | PE teachers caring teaching behaviors | NA |
| 33 | Mordal-Moen & Green | 2014 | 2 | Norway | Qualitative | NA | PE student teachers' teaching styles | NA |
| 34 | Konstantinidou & Zisi | 2017 | 2 | Greece | Qualitative | NA | PE Teacher teaching behaviors and skills | NA |
| 35 | Syrmpas et al. | 2017 | 4 | Greece | Qualitative | NA | PE teachers' experiences and beliefs concerning the production and the reproduction teaching approach | NA |
| 36 | Hovdal et al. | 2021 | 4 | Norway | Qualitative | Secondary | PE teachers teaching and management behaviors during PE class | Disruptive situations in PE class |
| 37 | Reuker | 2017 | 1 | Germany | Qualitative | NA | PE teachers teaching expertise | NA |
| 38 | Farias et al. | 2017 | 3 | Portugal | Qualitative | NA | Student teachers teaching behaviors | equity and inclusion behaviors in class |
| 39 | Aelterman et al. | 2014 | 5 | Belgium | Quantitative | Secondary | autonomy supportive behavior and structuring | NA |
| 40 | De Meyer et al. | 2014 | 8 | Belgium | Quantitative | Secondary | PE teachers controlling behaviors | Motivation for PE - autonomous, controlled motivations, and amotivation |
| 41 | Berghe et al. | 2015 | 5 | Belgium | Quantitative | Secondary | PE teachers behaviors | Student engagement and disengagement |
| 42 | Kokkonen et al. | 2013 | 4 | Finland | Quantitative | Secondary | PE teachers democratic and social support behaviors | Task and Ego Orientation in PE |
| 43 | Burgueno & Medina-Casaubon | 2021 | 2 | Spain | Quantitative | Secondary | PE teachers interpersonal behaviors | Satisfaction in PE |
| 44 | Escriva-Boulley et al. | 2018 | 4 | France | Quantitative | Elementary | PE Teachers' motivating style | PE students physical activity levels (MVPA) using accelerometers |
| 45 | Gonzalez-Peno et al. | 2021 | 3 | Spain | Quantitative | DNM | PE teachers need-supporttive and need-thwarting teaching behaviors | PE students behavioral engagement in PE |
| 46 | den Berghe et al. | 2013 | 7 | Belgium | Quantitative | NA | PE teachers teaching behavior | NA |
| 47 | Tessier et al. | 2010 | 3 | France | Quantitative | Secondary | PE teachers teaching behaviors | Motivation toward PE |
| 48 | Moe et al. | 2022 | 3 | Italy | Quantitative | NA | PE teachers own perception of Basic Need satisfaction and frustration | NA |
| 49 | Escriva-Boulley et al. | 2021 | 4 | France | Quantitative | NA | PE teachers own perception of motivating (teaching) style | NA |
| 50 | Burel et al. | 2021 | 3 | France | Quantitative | NA | PE teachers own perception of subjective feelings and motivational styles | NA |
| 51 | Schnitzius et al. | 2021 | 5 | Germany | Quantitative | NA | PE teachers own perception of teaching behaviors | NA |
| 52 | Coteron et al. | 2020 | 4 | Spain | Quantitative | Secondary | PE teachers OWN perception of their Basic needs satisfaction | Behaviorsal engagement - effort, attention and persistence in PE class |
| 53 | Hein et al. | 2018 | 3 | Estonia | Quantitative | Secondary | PE Teachers perception of their controlling behaviors | NA |
| 54 | Franco et al. | 2023 | 3 | Spain | Quantitative | NA | PE teachers’ feasibility beliefs to implement motivational strategies. | NA |
| 55 | Digelidis et al. | 2003 | 2 | Estonia | Quantitative | Secondary | PE teachers perceived feedback | Intrinsic motivation for PE |
| 56 | Syrmpas et al. | 2015 | 3 | Greece | Quantitative | NA | PE teachers perception of Spectrum teaching style | NA |
| 57 | Franco et al. | 2023 | 4 | Spain | Quantitative | NA | PE teachers psychological autonomy support and structure teaching style | Satisfaction and frustration in PE |
| 58 | Cronin et al. | 2018 | 4 | UK | Quantitative | Secondary | PE teachers' teaching climate - teaching autonomy support | Perceived Life Skills Development through PE |
| 59 | Koka | 2010 | 1 | Estonia | Quantitative | Secondary | PE techer teaching behaviors | Student competence in PE |
| 60 | Van Doren et al. | 2021 | 6 | Belgium | Quantitative | Secondary | perceived (de)motivating teaching style of PE teachers | motivation for PE |
| 61 | Vlachos & Papaioannou | 2023 | 2 | Greece | Quantitative | Elementary  Secondary | perceived behavioral strategies of PE teachers | Satisfaction in PE lesson |
| 62 | Haerens et al. | 2015 | 5 | Belgium | Quantitative | Secondary | Perceived behaviors of PE teachers | Satisfaction and frustration in PE |
| 63 | Cocca et al. | 2023 | 6 | Austria | Quantitative | Tertiary | perceived PE teacher behaviors | NA |
| 64 | Moreno-Casado et al. | 2022 | 6 | Spain | Quantitative | Secondary | perceived PE teacher leadership | Satisfaction in PE lesson |
| 65 | De Meyer et al. | 2016 | 6 | Belgium | Quantitative | Secondary | Perceived PE teacher teaching behaviors -autonomy supportive or controlling | Motivation toward PE |
| 66 | Sanchez-Oliva et al. | 2017 | 5 | Spain | Quantitative | Secondary | perceived PE teachers autonomy support ,structure, and relatedness support | Needs satisfaction in PE |
| 67 | Kalajas-Tilga et al. | 2020 | 5 | Estonia | Quantitative | Secondary | Perceived PE Teachers autonomy support behavior | Needs satisfaction in PE - autonomy, competence relatedness |
| 68 | Tilga et al. | 2020 | 4 | Estonia | Quantitative | Secondary | Perceived PE teachers autonomy supportive behaviors | Satisfaction and frustration in PE |
| 69 | Leyton-Roman et al. | 2020 | 3 | Spain | Quantitative | Secondary | perceived PE teachers autonomy support | Needs satisfaction in PE - autonomy, competence relatedness |
| 70 | Zimmermann et al. | 2020 | 4 | Germany | Quantitative | Elementary  Secondary | perceived PE teachers autonomy-support teaching | PE students academic self-efficacy in PE |
| 71 | Cronin et al. | 2019 | 7 | UK | Quantitative | Secondary | perceived PE teachers autonomy-supportive and controlling teaching | Satisfaction and frustration in PE |
| 72 | Tilga et al. | 2020 | 5 | Estonia | Quantitative | Secondary | perceived PE teachers autonomy-supportive behavior | Satisfaction and frustration in PE |
| 73 | Tilga et al. | 2020 | 5 | Estonia | Quantitative | Secondary | perceived PE teachers autonomy-supportive behavior | Needs satisfaction in PE |
| 74 | Tilga et al. | 2021 | 4 | Estonia | Quantitative | Secondary | Perceived PE teachers autonomy-supportive behavior | Satisfaction and frustration in PE |
| 75 | Leisterer & Paschold | 2022 | 2 | Germany | Quantitative | Secondary | perceived PE teachers autonomy-supportive teaching | Enjoyment in PE - pleasure, flow, relaxation |
| 76 | Diloy-Pena et al. | 2021 | 5 | Spain | Quantitative | Secondary | perceived PE teachers Basic Psychological Needs Support | Satisfaction towards PE |
| 77 | Leyton-Roman et al. | 2021 | 4 | Spain | Quantitative | Secondary | perceived PE teachers controlling behaviors | Motivation in PE |
| 78 | Tilga et al. | 2019 | 5 | Estonia | Quantitative | Secondary | Perceived PE teachers controlling behaviors | Frustrations in PE |
| 79 | Emeljanovas et al. | 2020 | 6 | Lithuania | Quantitative | Secondary | Perceived PE teachers controlling behaviors | Students Physical Activity during PE class using accelerometers |
| 80 | Abos et al. | 2021 | 4 | Spain | Quantitative | Secondary | perceived PE teachers controlling teaching behaviors | Frustrations in PE |
| 81 | Burgueno et al. | 2021 | 5 | Spain | Quantitative | Secondary | perceived PE teachers controlling teaching behaviors | Needs satisfaction in PE |
| 82 | Leo et al. | 2023 | 5 | Spain | Quantitative | Elementary  Secondary | perceived PE teachers interpersonal relatedness teaching style | Relatedness satisfaction in PE |
| 83 | Leo et al. | 2022 | 5 | Spain | Quantitative | Elementary  Secondary | perceived PE teachers' interpersonal styles | PE engagement - behavioral engagement, emotional engagement |
| 84 | Manzano-Sanchez | 2022 | 1 | Spain | Quantitative | Secondary | perceived PE teachers' motivational climate | Personal and Social Responsibility -how students normally behave |
| 85 | Sanchez -Oliva et al. | 2014 | 5 | Spain | Quantitative | Secondary | perceived PE teachers autonomy, competence and relatedness support | Needs satisfaction in PE |
| 86 | Sanchez-Oliva et al. | 2014 | 5 | Spain | Quantitative | Secondary | perceived PE teachers autonomy, competence and relatedness support | Needs satisfaction in PE |
| 87 | Sanchez-Oliva et al. | 2020 | 6 | Spain | Quantitative | Secondary | perceived PE teachers autonomy, competence and relatedness support | Needs satisfaction in PE |
| 88 | Castillo et al. | 2020 | 5 | Spain | Quantitative | Secondary | perceived PE teachers transformational teaching | Passion towards PE |
| 89 | Koka et al. | 2019 | 5 | Estonia | Quantitative | Elementary  Secondary | Perceived PE Teachers controlling behaviors | Frustrations in PE |
| 90 | Koka & Hein | 2003 | 4 | Greece | Quantitative | Secondary | Perceived PE teachers-initiated motivational climate in PE | Goal orientation in PE - Task and Ego |
| 91 | Jowett et al. | 2023 | 4 | UK | Quantitative | Secondary | perceived PE teachers-student relationship | Intrinsic motivation for PE |
| 92 | Leo et al. | 2022 | 5 | Spain | Quantitative | Elementary  Secondary | perceived teaching behavior | Students Need Satisfaction |
| 93 | Viksi & Tilga | 2022 | 2 | Estonia | Quantitative | Elementary  Secondary | Perceived PE teachers internal and external controlling behaviors | Frustrations in PE |
| 94 | Syrmpas et al. | 2020 | 5 | Greece | Quantitative | NA | Preservice PE teacher perception of Spectrum teaching style | NA |
| 95 | Verma et al. | 2019 | 5 | UK | Quantitative | Secondary | Teachers' Transformational Teaching | Self-presentation motives for physical activity |
| 96 | Sanchez et al. | 2012 | 3 | USA | Mixed method | Tertiary | PE teacher teaching style and teaching style preference | Perceived exertion (RPE) |
| 97 | Hastie et al. | 2016 | 3 | USA | Mixed method | NA | PE teacher's mastery climate in PE | Student engagement - observation |
| 98 | Glotova & Hastie | 2014 | 2 | USA | Mixed method | Tertiary | preservice PE teachers teaching style belief | NA |
| 99 | Wright et al. | 2009 | 3 | USA | Mixed method | Elementary  Secondary | Student teacher and PE teachers' personal perception about teaching, learning and mentoring | NA |
| 100 | Jin & Yun | 2013 | 2 | USA | Mixed methods | Secondary | PE teacher Physical Activity Promotion during PE behavior | implementation intentions |
| 101 | Kim & Housner | 2010 | 2 | USA | Mixed methods | Elementary | PE teachers behaviors - planning,decision making, and behaviors | Student behaviors during PE |
| 102 | Schwamberger & Curtner-Smith | 2018 | 2 | USA | Qualitative | NA | Preservice teacher's teaching behaviors | NA |
| 103 | Germain | 2010 | 1 | USA | Qualitative | NA | Kinesiology and PE instructors use of various leadership behaviors | NA |
| 104 | Hannon & Ratliffe | 2007 | 2 | USA | Qualitative | NA | PE teachers' teaching behaviors | NA |
| 105 | Larson & Silvermann | 2005 | 2 | USA | Qualitative | NA | PE teacher caring behaviors | NA |
| 106 | Sinelnikov & Hastie | 2010 | 2 | USA | Qualitative | NA | PE teacher motivational climate in volleyball | NA |
| 107 | Wilkinson et al. | 2013 | 5 | Canada | Qualitative | NA | PE teacher personal teaching behaviors | NA |
| 108 | Zeng et al. | 2009 | 4 | USA | Qualitative | Secondary | PE teacher teaching behaviors | learning in PE |
| 109 | Wright & Craig | 2011 | 2 | USA | Qualitative | Elementary | PE teachers behaviors and teaching strategies in PE class | personal and social responsibility behaviors |
| 110 | Jenkins et al. | 2006 | 4 | USA | Qualitative | NA | perceived behaviors of preservice PE teachers | NA |
| 111 | Larson | 2006 | 1 | USA | Qualitative | Elementary  Secondary | perceived PE teacher caring behaviors | NA |
| 112 | Jenkins et al. | 2006 | 4 | USA | Qualitative | NA | perception about PE and PE teacher effective teaching behaviors | NA |
| 113 | Sato & Haegele | 2017 | 2 | USA | Qualitative | NA | preservice adapted PE teachers perceptions of teaching | NA |
| 114 | Larson | 2005 | 1 | USA | Qualitative | NA | Preservice teachers experience-teaching styles | NA |
| 115 | Norris et al. | 2017 | 5 | USA | Qualitative | NA | PE Teacher teaching quality evaluation - behavior and effectiveness | NA |
| 116 | Lavay et al. | 2014 | 3 | USA | Quantitative | NA | Adapted PE teachers teaching styles | NA |
| 117 | Shen et al. | 2012 | 5 | USA | Quantitative | Secondary | observed PE teachers relatedness support | autonomy in PE |
| 118 | Otundo & Garn | 2019 | 2 | USA | Quantitative | Secondary | Observed PE teachers supportive teachig | situational interest |
| 119 | Webster et al. | 2012 | 3 | USA | Quantitative | NA | PE teachers own perception of teaching PE to students | NA |
| 120 | Lavay et al. | 2012 | 4 | USA | Quantitative | NA | PE teachers own perception of teaching practices | NA |
| 121 | Hines & Paulson | 2007 | 2 | USA | Quantitative | NA | PE teachers' own perception of their teaching behaviors | classic/conforming and positive behaviors- conflict, moodiness, and risk-taking |
| 122 | Kulinna & Cothran | 2003 | 2 | USA | Quantitative | NA | PE teachers' own perception of their teaching styles | NA |
| 123 | Bolter et al. | 2018 | 3 | USA | Quantitative | Secondary | PE teachers teaching behaviors | Comparisons |
| 124 | Maldonado et al. | 2019 | 5 | Mexico | Quantitative | Secondary | perceived PE teacher autonomy support in PE class | Motivation in PE |
| 125 | Ullrich-French & Cox | 2014 | 2 | USA | Quantitative | Secondary | Perceived PE teacher support | Motivation regulations in PE setting |
| 126 | Cox et al. | 2011 | 4 | USA | Quantitative | Secondary | Perceived PE Teacher support | situation specific anxiety - social evaluation of one's body during PE class |
| 127 | Cox & Ullrich-French | 2010 | 2 | USA | Quantitative | Secondary | Perceived PE teacher support | Peer acceptance and physical competence in PE |
| 128 | Cox et al. | 2009 | 3 | USA | Quantitative | Secondary | Perceived PE teacher support | Peer acceptance in PE |
| 129 | Cox & Williams | 2008 | 2 | USA | Quantitative | Elementary | Perceived PE teachers support | Physical competence in PE class |
| 130 | Beauchamp et al. | 2010 | 6 | Canada | Quantitative | Secondary | perceived PE teachers transformational teaching behaviors | Motivation in PE class |
| 131 | Bourne et al. | 2015 | 6 | Canada | Quantitative | Secondary | Perceived transformational teaching of PE teachers | Self efficacy beliefs |
| 132 | Todorovich | 2009 | 1 | USA | Quantitative | NA | Preservice PE teachers teaching behaviors with high ego orientation | NA |
| 133 | Zeng | 2016 | 1 | USA | Quantitative | Tertiary | student teacher PETE major students | NA |
| 134 | Karakaya et al. | 2022 | 3 | Turkey | Mixed method | NA | PE teachers' own perception of their cultural capital competencies | NA |
| 135 | Telford et al. | 2021 | 5 | Australia | Mixed method | NA | PE teachers teaching style | NA |
| 136 | Sparks et al. | 2015 | 5 | Australia | Qualitative | Secondary | PE teacher relatedness supportive behaviors | NA |
| 137 | Nasri et al. | 2022 | 4 | Tunisia | Qualitative | NA | PE teachers teaching behaviors (verbal and nonverbal) | NA |
| 138 | Razouki et al. | 2021 | 4 | Morocco | Qualitative | NA | PE teachers teaching practices | NA |
| 139 | Ferraz et al. | 2021 | 3 | Brazil | Qualitative | NA | Preservice PE teacher experieces | NA |
| 140 | Fyall & Metzler | 2019 | 2 | New Zealand | Qualitative | Tertiary | Preservice PE teachers teaching styles to implement new PE curriculum | NA |
| 141 | Parsak & Sarac | 2019 | 2 | Turkey | Quantitative | NA | PE teachers self-reported and observed and teaching styles | NA |
| 142 | Bechter et al. | 2019 | 3 | Australia | quantitative | Secondary | PE teachers observed student-centered instructional behaviors | Motivation for PE |
| 143 | Abdulla et al. | 2022 | 4 | Australia | Quantitative | Elementary  Secondary | PE teachers own perceived need support | Satisfaction and frustration in PE |
| 144 | Ada et al. | 2021 | 5 | Turkey | Quantitative | NA | PE teacher's own perception of teacher-student relationship | NA |
| 145 | Tenorio et al. | 2021 | 4 | Brazil | Quantitative | NA | PE teachers own perception of teaching PE content and their teaching style to HS students | Motivation to participate during PE class |
| 146 | Jackson et al. | 2012 | 5 | Australia | Quantitative | Secondary | Perceived PE teachers behaviors | Level of effort in PE |
| 147 | Sparks et al. | 2016 | 4 | Australia | Quantitative | Secondary | perceived PE teachers individualized conversations - communication teaching style | Motivation to participate in PE |
| 148 | Sparks et al. | 2017 | 4 | Australia | Quantitative | Secondary | perceived PE teachers relatedness support behaviors | Enjoyment in PE |
| 149 | Gairns et al. | 2015 | 3 | Australia | Quantitative | Secondary | PE teachers interpersonal behaviors | Relation-inferred - self-efficacy in students |
| 150 | Moy et al. | 2014 | 3 | Australia | Quantitative | Tertiary | preservice PE teachers teaching approaches | Predominant teaching method used by teacher |

NA = not applicable; DNM = did not mention

References of all included studies

Abdulla, A., Whipp, P. R., McSporran, G., & Teo, T. (2022). An interventional study with the Maldives generalist teachers in primary school physical education: An application of self-determination theory. PloS one, 17(5), e0268098. https://doi.org/10.1371/journal.pone.0268098

Abós, Á., Burgueño, R., García-González, L., & Sevil-Serrano, J. (2021). Influence of internal and external controlling teaching behaviors on students’ motivational outcomes in physical education: Is there a gender difference?. J. Teach. Phys. Educ. 41(3), 502-512. https://doi.org/10.1123/jtpe.2020-0316

Ada, E. N., Ahmad, H., Uzun, N. B., Jowett, S., & Kazak, Z. (2021). Cross-cultural adaptation of the Turkish and Kuwaiti teacher–student relationship questionnaire in physical education (TSRQ-PE Teacher Version): Testing for measurement invariance. Sustainability, 13(3), 1387. https://doi.org/10.3390/su13031387

Aelterman, N., Vansteenkiste, M., Van den Berghe, L., De Meyer, J., & Haerens, L. (2014). Fostering a need-supportive teaching style: Intervention effects on physical education teachers’ beliefs and teaching behaviors. J. Sport Exerc. Psychol. 36(6), 595-609. 10.1123/jsep.2013-0229

Beauchamp, M. R., Barling, J., Li, Z., Morton, K. L., Keith, S. E., & Zumbo, B. D. (2010). Development and psychometric properties of the transformational teaching questionnaire. J. Health Psychol. 15(8), 1123-1134. https://doi.org/10.1177/1359105310364175

Bechter, B. E., Dimmock, J. A., & Jackson, B. (2019). A cluster-randomized controlled trial to improve student experiences in physical education: Results of a student-centered learning intervention with high school teachers. Psychol. Sport Exerc. 45, 101553. https://doi.org/10.1016/j.psychsport.2019.101553

Behzadnia, B. (2021). The relations between students’ causality orientations and teachers’ interpersonal behaviors with students’ basic need satisfaction and frustration, intention to physical activity, and well-being. Phys. Educ. Sport Pedagogy. 26(6), 613-632. https://doi.org/10.1080/17408989.2020.1849085

Behzadnia, B., Adachi, P. J., Deci, E. L., & Mohammadzadeh, H. (2018). Associations between students' perceptions of physical education teachers' interpersonal styles and students' wellness, knowledge, performance, and intentions to persist at physical activity: A self-determination theory approach. Psychol. Sport Exerc. 39, 10-19. https://doi.org/10.1016/j.psychsport.2018.07.003

Behzadnia, B., Rezaei, F., & Salehi, M. (2022). A need-supportive teaching approach among students with intellectual disability in physical education. Psychol. Sport Exerc. 60, 102156. https://doi.org/10.1016/j.psychsport.2022.102156

Bolter, N. D., Kipp, L., & Johnson, T. (2018). Teaching sportsmanship in physical education and youth sport: comparing perceptions of teachers with students and coaches with athletes. J. Teach. Phys. Educ. 37(2). https://doi.org/10.1123/jtpe.2017-0038

Bourne, J., Liu, Y., Shields, C. A., Jackson, B., Zumbo, B. D., & Beauchamp, M. R. (2015). The relationship between transformational teaching and adolescent physical activity: The mediating roles of personal and relational efficacy beliefs. J. Health Psychol. 20(2), 132-143. https://doi.org/10.1177/1359105313500096

Burel, N., Tessier, D., & Langdon, J. (2021). Are teachers’ subjective feelings linked with need-supportive and need-thwarting motivating styles? A cross-lagged pilot study in physical education. Eur. J. Psychol. Educ. 36(4), 1221-1241. https://doi.org/10.1007/s10212-020-00517-x

Burgueño, R., & Medina-Casaubón, J. (2021). Validity and reliability of the interpersonal behaviors questionnaire in physical education with Spanish secondary school students. Percept. Mot. Skills. 128(1), 522-545. https://doi.org/10.1177/0031512520948286

Burgueño, R., Abós, Á., García-González, L., Tilga, H., & Sevil-Serrano, J. (2021). Evaluating the psychometric properties of a scale to measure perceived external and internal faces of controlling teaching among students in physical education. Int. J. Environ. Res. Public Health. 18(1), 298. https://doi.org/10.3390/ijerph18010298

Burhan, P., & Leyla, S. (2020). Turkish Physical Education Teachers’ Use of Teaching Styles: Self-Reported Versus Observed. J. Teach. Phys. Educ. 39, 137-146. https://doi.org/10.1123/jtpe.2018-0320

Castillo, I., Molina-García, J., Estevan, I., Queralt, A., & Álvarez, O. (2020). Transformational teaching in physical education and students’ leisure-time physical activity: The mediating role of learning climate, passion and self-determined motivation. Int. J. Environ. Res. Public Health. 17(13), 4844. https://doi.org/10.3390/ijerph17134844

Cheon, S. H., & Reeve, J. (2013). Do the benefits from autonomy-supportive PE teacher training programs endure?: A one-year follow-up investigation. Psychol. Sport Exerc. 14(4), 508-518. https://doi.org/10.1016/j.psychsport.2013.02.002

Cheon, S. H., Reeve, J., & Moon, I. S. (2012). Experimentally based, longitudinally designed, teacher-focused intervention to help physical education teachers be more autonomy supportive toward their students. J. Sport Exerc. Psychol. 34(3), 365-396. https://doi.org/10.1123/jsep.34.3.365

Cheon, S. H., Reeve, J., & Ntoumanis, N. (2018). A needs-supportive intervention to help PE teachers enhance students' prosocial behavior and diminish antisocial behavior. Psychol. Sport Exerc. 35, 74-88. https://doi.org/10.1016/j.psychsport.2017.11.010

Cheon, S. H., Reeve, J., & Ntoumanis, N. (2019). An intervention to help teachers establish a prosocial peer climate in physical education. Learn. Instruct. 64, 101223. https://doi.org/10.1016/j.learninstruc.2019.101223

Cheon, S. H., Reeve, J., & Song, Y. G. (2019). Recommending goals and supporting needs: An intervention to help physical education teachers communicate their expectations while supporting students’ psychological needs. Psychol. Sport Exerc. 41, 107-118. https://doi.org/10.1016/j.psychsport.2018.12.008

Cheon, S. H., Reeve, J., Marsh, H. W., & Song, Y. G. (2022). Intervention-enabled autonomy-supportive teaching improves the PE classroom climate to reduce antisocial behavior. Psychol. Sport Exerc. 60, 102174. https://doi.org/10.1016/j.psychsport.2022.102174

Choi, S. M., Sum, R. K. W., Wallhead, T., Ha, A. S. C., Sit, C. H. P., Shy, D. Y., & Wei, F. M. (2020). Preservice physical education teachers’ perceived physical literacy and teaching efficacy. J. Teach. Phys. Educ. 40(1), 146-156. https://doi.org/10.1123/jtpe.2019-0076

Cocca, A., Veulliet, N., Drenowatz, C., Wirnitzer, K., Greier, K., & Ruedl, G. (2023). Assessment of a Novel Instrument Measuring Perceived Physical Education Teachers’ In-Class Skills. Behav. Sci. 13(1), 42. https://doi.org/10.3390/bs13010042

Coterón, J., Franco, E., Ocete, C., & Pérez-Tejero, J. (2020). Teachers’ psychological needs satisfaction and thwarting: Can they explain students’ behavioural engagement in physical education? A multi-level analysis. Int. J. Environ. Res. Public Health. 17(22), 8573. https://doi.org/10.3390/ijerph17228573

Cox, A. E., & Ullrich-French, S. (2010). The motivational relevance of peer and teacher relationship profiles in physical education. Psychol. Sport Exerc. 11(5), 337-344. https://doi.org/10.1016/j.psychsport.2010.04.001

Cox, A. E., Ullrich-French, S., Madonia, J., & Witty, K. (2011). Social physique anxiety in physical education: Social contextual factors and links to motivation and behavior. Psychol. Sport Exerc. 12(5), 555-562. https://doi.org/10.1016/j.psychsport.2011.05.001

Cox, A., & Williams, L. (2008). The roles of perceived teacher support, motivational climate, and psychological need satisfaction in students’ physical education motivation. J. Sport Exerc. Psychol. 30(2), 222-239. https://doi.org/10.1123/jsep.30.2.222

Cox, A., Duncheon, N., & McDavid, L. (2009). Peers and teachers as sources of relatedness perceptions, motivation, and affective responses in physical education. Res. Q. Exerc. Sport. 80(4), 765-773. 10.1080/02701367.2009.10599618

Cronin, L. D., Allen, J., Mulvenna, C., & Russell, P. (2018). An investigation of the relationships between the teaching climate, students’ perceived life skills development and well-being within physical education. Phys. Educ. Sport Pedagogy. 23(2), 181-196. https://doi.org/10.1080/17408989.2017.1371684

Cronin, L., Marchant, D., Allen, J., Mulvenna, C., Cullen, D., Williams, G., & Ellison, P. (2019). Students’ perceptions of autonomy-supportive versus controlling teaching and basic need satisfaction versus frustration in relation to life skills development in PE. Psychol. Sport Exerc. 44, 79-89. https://doi.org/10.1016/j.psychsport.2019.05.003

De Meyer, J., Soenens, B., Vansteenkiste, M., Aelterman, N., Van Petegem, S., & Haerens, L. (2016). Do students with different motives for physical education respond differently to autonomy-supportive and controlling teaching?. Psychol. Sport Exerc. 22, 72-82. https://doi.org/10.1016/j.psychsport.2015.06.001

De Meyer, J., Tallir, I. B., Soenens, B., Vansteenkiste, M., Aelterman, N., Van den Berghe, L., ... & Haerens, L. (2014). Does observed controlling teaching behavior relate to students’ motivation in physical education?. J. Educ. Psychol. 106(2), 541. 10.1037/a0034399

Digelidis, N., Papaioannou, A., Laparidis, K., & Christodoulidis, T. (2003). A one-year intervention in 7th grade physical education classes aiming to change motivational climate and attitudes towards exercise. Psychol. Sport Exerc. 4(3), 195-210. https://doi.org/10.1016/S1469-0292(02)00002-X

Diloy-Peña, S., García-González, L., Sevil-Serrano, J., Sanz-Remacha, M., & Abós, A. (2021). Motivating teaching style in Physical Education: how does it affect the experiences of students. Apunts. Educ. Fís. 144, 44-51. https://doi.org/10.5672/apunts.2014-0983.es.(2021/2).144.06

Emeljanovas, A., Mieziene, B., Putriute, V., Sinkariova, L., Tilindiene, I., & Trinkuniene, L. (2020). The Relationship Between Objectively Measured Class Physical Activity and Teachers' Autonomy Supportive and Controlling Behaviors. Rev. Psicol. Deporte. 29. https://hdl.handle.net/20.500.12259/129765

Escriva-Boulley, G., Haerens, L., Tessier, D., & Sarrazin, P. (2021). Antecedents of primary school teachers’ need-supportive and need-thwarting styles in physical education. Eur. Phys. Educ. Rev. 27(4), 961-980. https://doi.org/10.1177/1356336X21100462

Escriva-Boulley, G., Tessier, D., Ntoumanis, N., & Sarrazin, P. (2018). Need-supportive professional development in elementary school physical education: Effects of a cluster-randomized control trial on teachers’ motivating style and student physical activity. Sport Exerc. Perform. Psychol. 7(2), 218. 10.1037/spy0000119

Farias, C., Hastie, P. A., & Mesquita, I. (2017). Towards a more equitable and inclusive learning environment in sport education: Results of an action research-based intervention. Sport Educ. Soc. 22(4), 460-476. https://doi.org/10.1080/13573322.2015.1040752

Ferraz, O. L., Vidoni, C., & Boas, M. V. (2021). Bridging the gap between theory and practice: the impact of school–university partnership in a PETE program. Sport Educ. Soc. 26(7), 788-799. https://doi.org/10.1080/13573322.2020.1851182

Franco, E., González-Peño, A., & Coterón, J. (2023). Understanding physical education teachers’ motivational outcomes and feasibility beliefs to implement motivational strategies: The role of perceived pressures from a variable-and person-centered approach. Psychol. Sport Exerc. 64, 102337. https://doi.org/10.1016/j.psychsport.2022.102337

Franco, E., González-Peño, A., Trucharte, P., & Martínez-Majolero, V. (2023). Challenge-based learning approach to teach sports: Exploring perceptions of teaching styles and motivational experiences among student teachers. J. Hosp. Leis. Sport Tour. Educ. 32, 100432. https://doi.org/10.1016/j.jhlste.2023.100432

Fyall, G., & Metzler, M. W. (2019). Aligning critical physical education teacher education and models-based practice. Phys. Educat. 76(1), 24-56. 10.18666/TPE-2019-V76-I1-8370

Gairns, F., Whipp, P. R., & Jackson, B. (2015). Relational perceptions in high school physical education: Teacher-and peer-related predictors of female students’ motivation, behavioral engagement, and social anxiety. Front. Psychol. 6, 140807. https://doi.org/10.3389/fpsyg.2015.00850

Germain, J. L. (2010). Physical program leadership: from kinesiology in the classroom to fitness training in the field. Quest, 62(3), 287-295. https://doi.org/10.1080/00336297.2010.10483649

Girard, A., Gal-Petitfaux, N., & Vors, O. (2022). The student's experience of teacher support in French vocational high-school classes with difficulties in school engagement in physical education: interest of mixed methods research. Phys. Educ. Sport Pedagogy. 27(2), 156-171. https://doi.org/10.1080/17408989.2021.1999918

Glotova, O. N., & Hastie, P. A. (2014). Learning to teach Sport Education in Russia: factors affecting model understanding and intentions to teach. Sport Educ. Soc. 19(8), 1072-1088. https://doi.org/10.1080/13573322.2012.732567

González-Peño, A., Franco, E., & Coterón, J. (2021). Do observed teaching behaviors relate to students’ engagement in physical education?. Int. J. Environ. Res. Public Health. 18(5), 2234. https://doi.org/10.3390/ijerph18052234

Haerens, L., Aelterman, N., Van den Berghe, L., De Meyer, J., Soenens, B., & Vansteenkiste, M. (2013). Observing physical education teachers’ need-supportive interactions in classroom settings. J. Sport Exerc. Psychol. 35(1), 3-17. https://doi.org/10.1123/jsep.35.1.3

Haerens, L., Aelterman, N., Vansteenkiste, M., Soenens, B., & Van Petegem, S. (2015). Do perceived autonomy-supportive and controlling teaching relate to physical education students' motivational experiences through unique pathways? Distinguishing between the bright and dark side of motivation. Psychol. Sport Exerc. 16, 26-36. https://doi.org/10.1016/j.psychsport.2014.08.013

Hannon, J. C., & Ratliffe, T. (2007). Opportunities to participate and teacher interactions in coed versus single-gender physical education settings. Phys. Educat. 64(1), 11.

Hastie, P. A., Rudisill, M. E., & Boyd, K. (2016). An ecological analysis of a preschool mastery climate physical education programme. Phys. Educ. Sport Pedagogy. 21(2), 217-232. https://doi.org/10.1080/17408989.2015.1017454

Hein, V., Emeljanovas, A., & Mieziene, B. (2018). A cross-cultural validation of the controlling teacher behaviours scale in physical education. Eur. Phys. Educ. Rev. 24(2), 209-224. https://doi.org/10.1177/1356336X16681

Hines, A. R., & Paulson, S. E. (2006). Parents' and teachers' perceptions of adolescent storm and stress: Relations with parenting and teaching styles. Adolescence, 41(164).

Hosseini, F. B., Ghorbani, S., & Rezaeeshirazi, R. (2020). Effects of perceived autonomy support in the physical education on basic psychological needs satisfaction, intrinsic motivation and intention to perform physical activity in high school students. Int. J. Sch. Health. 7(4), 39-46. https://doi.org/10.30476/intjsh.2020.88171.1106

Hovdal, D. O. G., Larsen, I. B., Haugen, T., & Johansen, B. T. (2021). Understanding disruptive situations in physical education: Teaching style and didactic implications. Eur. Phys. Educ. Rev. 27(3), 455-472. https://doi.org/10.1177/1356336X209604

Hsu, W. T., & Pan, M. (2019). Development and validation of the teacher RISE support scale in physical education. J. Teach. Phys. Educ. 38(4), 286-295. https://doi.org/10.1123/jtpe.2018-0245

Hsu, W. T., Shang, I. W., Pan, Y. H., & Chou, C. C. (2023). Students’ efficacy profiles and outcomes of perceived relation-inferred self-efficacy support in physical education. International J. Sport Exerc. Psychol. 21(1), 56-69. https://doi.org/10.1080/1612197X.2022.2043926

Jackson, B., Whipp, P. R., Chua, K. P., Pengelley, R., & Beauchamp, M. R. (2012). Assessment of tripartite efficacy beliefs within school-based physical education: Instrument development and reliability and validity evidence. Psychol. Sport Exerc. 13(2), 108-117. https://doi.org/10.1016/j.psychsport.2011.10.007

Jenkins, J. M., Jenkins, P., Collums, A., & Werhonig, G. (2006). Student perceptions of a conceptual physical education activity course. Phys. Educat. 63(4), 210.

Jin, J., & Yun, J. (2013). Three frameworks to predict physical activity behavior in middle school inclusive physical education: A multilevel analysis. Adapt. Phys. Activ. Q. 30(3), 254-270. https://doi.org/10.1123/apaq.30.3.254

Jowett, S., Warburton, V. E., Beaumont, L. C., & Felton, L. (2023). Teacher–Student relationship quality as a barometer of teaching and learning effectiveness: Conceptualization and measurement. Br. J. Educ. Psychol. 93(3), 842-861. https://doi.org/10.1111/bjep.12600

Jung, H., & Choi, E. (2016). The importance of indirect teaching behaviour and its educational effects in physical education. Phys. Educ. Sport Pedagogy. 21(2), 121-136. https://doi.org/10.1080/17408989.2014.923990

Kahan, D., Sinclair, C., Saucier Jr, L., & NguyenCaiozzi, N. (2003). Feedback profiles of cooperating teachers supervising the same student teacher. Phys. Educat. 60(4), 180.

Kalajas-Tilga, H., Koka, A., Hein, V., Tilga, H., & Raudsepp, L. (2020). Motivational processes in physical education and objectively measured physical activity among adolescents. J. Sport Health Sci. 9(5), 462-471. https://doi.org/10.1016/j.jshs.2019.06.001

Karakaya, Y. E., Bingölbali, A., & Şahin, A. (2022). Cultural capital competencies of teachers in sports education: A mixed-method study. Biomed. Hum. Kinet. 14(1), 191-203. 10.2478/bhk-2022-0024

Kim, H. Y., & Housner, L. D. (2010). The Influence of Class Size on the Planning Decision Making, Concerns, and Instructional Behaviors of Experienced and Inexperienced Teachers. Int. J. Appl. Sports Sci. 22(2). 10.24985/ijass.2010.22.2.77

Koka, A. (2010). The effect of age on relationships between perceived teaching behaviours, basic psychological needs and self-determined motivation in physical education. Acta Kinesiol. Univ. Tartu. 15, 23-34. 10.12697/akut.2010.15.02

KOKA, A. (2020). How teachers’ controlling behaviour can ruin students’ intrinsic motivation in a physical education lesson: Test of a conditional process model. Int. J. Sport Psychol. 51, 81-99. 10.7352/IJSP.2020.51.081

Koka, A., & Hein, V. (2003). Perceptions of teacher’s feedback and learning environment as predictors of intrinsic motivation in physical education. Psychol. Sport Exerc. 4(4), 333-346. https://doi.org/10.1016/S1469-0292(02)00012-2

Koka, A., Tilga, H., Kalajas-Tilga, H., Hein, V., & Raudsepp, L. (2019). Perceived controlling behaviors of physical education teachers and objectively measured leisure-time physical activity in adolescents. Int. J. Environ. Res. Public Health. 16(15), 2709. https://doi.org/10.3390/ijerph16152709

Kokkonen, J. A., Kokkonen, M. T., Telama, R. K., & Liukkonen, J. O. (2013). Teachers' behavior and pupils' achievement motivation as determinants of intended helping behavior in physical education. Scand. J. Educ. Res. 57(2), 199-216. https://doi.org/10.1080/00313831.2011.628692

Konstantinidou, E. P., & Zisi, V. Z. (2017). Do physical educators promote students’ creativity? an observational analysis study. Phys. Educat. 74(3). https://doi.org/10.18666/TPE-2017-V74-I3-7407

Kulinna, P. H., & Cothran, D. J. (2003). Physical education teachers’ self-reported use and perceptions of various teaching styles. Learn. Instr. 13(6), 597-609. https://doi.org/10.1016/S0959-4752(02)00044-0

Kwon, H. H., & Kim, M. (2010). Perceived leadership behavior of physical education teacher-coaches: When they teach vs. when they coach. J. Teach. Phys. Educ. 29(2), 131-145. https://doi.org/10.1123/jtpe.29.2.131

Lamb, C. A., Teraoka, E., Oliver, K. L., & Kirk, D. (2021). Pupils’ motivational and emotional responses to pedagogies of affect in physical education in Scottish secondary schools. Int. J. Environ. Res. Public Health. 18(10), 5183. https://doi.org/10.3390/ijerph18105183

Larson, A. (2005). Preservice teachers' field experience surprises: Some things never change. Phys. Educat. 62(3), 154.

Larson, A. (2006). Student perception of caring teaching in physical education. Sport Educ. Soc. 11(4), 337-352. https://doi.org/10.1080/13573320600924858

Larson, A., & Silverman, S. J. (2005). Rationales and practices used by caring physical education teachers. Sport Educ. Soc. 10(2), 175-193. https://doi.org/10.1080/13573320500111713

Lavay, B., Guthrie, S., & Henderson, H. (2014). The behavior management training and teaching practices of Nationally Certified Adapted Physical Education (CAPE) teachers. Palaestra, 28(1).

Lavay, B., Henderson, H., French, R., & Guthrie, S. (2012). Behavior management instructional practices and content of college/university physical education teacher education (PETE) programs. Phys. Educ. Sport Pedagogy. 17(2), 195-210. https://doi.org/10.1080/17408989.2010.548063

Leisterer, S., & Paschold, E. (2022). Increased perceived autonomy-supportive teaching in physical education classes changes students’ positive emotional perception compared to controlling teaching. Front. Psychol. 13, 1015362. https://doi.org/10.3389/fpsyg.2022.1015362

Leo, F. M., López-Gajardo, M. A., Rodríguez-González, P., Pulido, J. J., & Fernández-Río, J. (2023). How class cohesion and teachers’ relatedness supportive/thwarting style relate to students’ relatedness, motivation, and positive and negative outcomes in physical education. Psychol. Sport Exerc. 65, 102360. https://doi.org/10.1016/j.psychsport.2022.102360

Leo, F. M., Mouratidis, A., Pulido, J. J., López-Gajardo, M. A., & Sánchez-Oliva, D. (2022). Perceived teachers’ behavior and students’ engagement in physical education: The mediating role of basic psychological needs and self-determined motivation. Phys. Educ. Sport Pedagogy. 27(1), 59-76. https://doi.org/10.1080/17408989.2020.1850667

Leo, F. M., Pulido, J. J., Sánchez-Oliva, D., López-Gajardo, M. A., & Mouratidis, A. (2022). See the forest by looking at the trees: Physical education teachers’ interpersonal style profiles and students’ engagement. Eur. Phys. Educ. Rev. 28(3), 720-738. 10.1177/1356336X221075501

Leyton-Román, M., González-Vélez, J. J. L., Batista, M., & Jiménez-Castuera, R. (2020). Predictive model for amotivation and discipline in physical education students based on teaching–learning styles. Sustainability, 13(1), 187. https://doi.org/10.3390/su13010187

Leyton-Roman, M., Nunez, J. L., & Jimenez-Castuera, R. (2020). The importance of supporting student autonomy in physical education classes to improve intention to be physically active. Sustainability, 12(10), 4251. https://doi.org/10.3390/su12104251

Li, C., Kam, W. K. K., & Zhang, M. (2019). Physical Education Teachers' Behaviors and Intentions of Integrating STEM Education in Teaching. Phys. Educat. 76(4), 1086-1101. 10.18666/TPE-2019-V76-I4-9104

Li, S., Chen, L., Wu, Q., Xin, S., & Chen, Z. (2021). The behavior model of psychological contract between sports teachers and undergraduate students in China. Journal of Hospitality, Leisure, Sport & Tourism Education, 28, 100300.

Lim, B. C., & Wang, C. J. (2009). Perceived autonomy support, behavioural regulations in physical education and physical activity intention. Psychol. Sport Exerc. 10(1), 52-60. https://doi.org/10.1016/j.psychsport.2008.06.003

Liu, C. L., Ding, C. T., & Huang, Y. P. (2019). Teaching adapted physical education in the primary years–Taiwanese teachers’ attitudes. Sport Soc. 22(8), 1481-1496. https://doi.org/10.1080/17430437.2018.1529169

Liu, J. D., & Chung, P. K. (2017). Factor structure and measurement invariance of the Need-Supportive Teaching Style Scale for Physical Education. Percept. Mot. Skills. 124(4), 864-879. https://doi.org/10.1177/0031512517712803

Maldonado, E., Zamarripa, J., Ruiz-Juan, F., Pacheco, R., & Delgado, M. (2019). Teacher autonomy support in physical education classes as a predictor of motivation and concentration in Mexican students. Front.Psychol. 10, 471034. https://doi.org/10.3389/fpsyg.2019.02834

Manzano-Sánchez, D. (2022). Physical education classes and responsibility: The importance of being responsible in motivational and psychosocial variables. Int. J. Environ. Res. Public Health. 19(16), 10394. https://doi.org/10.3390/ijerph191610394

Manzano-Sanchez, D., Gomez-Marmol, A., & Valero-Valenzuela, A. (2020). Student and teacher perceptions of teaching personal and social responsibility implementation, academic performance and gender differences in secondary education. Sustainability, 12(11), 4590. https://doi.org/10.3390/su12114590

Moè, A., Consiglio, P., & Katz, I. (2022). Exploring the circumplex model of motivating and demotivating teaching styles: The role of teacher need satisfaction and need frustration. Teach. Teach. Educ. 118, 103823. https://doi.org/10.1016/j.tate.2022.103823

Moen, K. M., Westlie, K., Gerdin, G., Smith, W., Linnér, S., Philpot, R., ... & Larsson, L. (2020). Caring teaching and the complexity of building good relationships as pedagogies for social justice in health and physical education. Sport Educ. Soc. 25(9), 1015-1028. https://doi.org/10.1080/13573322.2019.1683535

Mordal-Moen, K., & Green, K. (2014). Physical education teacher education in Norway: The perceptions of student teachers. Sport Educ. Soc. 19(6), 806-823. https://doi.org/10.1080/13573322.2012.719867

Moreno-Casado, H., Leo, F. M., López-Gajardo, M. Á., García-Calvo, T., Cuevas, R., & Pulido, J. J. (2022). Teacher leadership and students’ psychological needs: A multilevel approach. Teach. Teach. Educ. 116, 103763. https://doi.org/10.1016/j.tate.2022.103763

Moy, B., Renshaw, I., & Davids, K. (2014). Variations in acculturation and Australian physical education teacher education students' receptiveness to an alternative pedagogical approach to games teaching. Phys. Educ. Sport Pedagogy. 19(4), 349-369. https://doi.org/10.1080/17408989.2013.780591

Nasri, B., Kadri, A., Souissi, N., & Rouissi, M. (2022). The effects of the socialization of physical education teachers on their modes of interaction with students in Tunisian schools. Front. Sociol. 6, 747092. https://doi.org/10.3389/fsoc.2021.747092

Norris, J. M., Kwon, J., van der Mars, H., & Kulinna, P. H. (2016). A document analysis of physical education teacher evaluation systems. Res. Q. Exerc. Sport. 87(S2), A130.

Otundo, J. O., & Garn, A. C. (2019). Student interest and engagement in middle school physical education: Examining the role of needs supportive teaching. Int. J. Educ. Psychol. 8(2), 137-161. 10.17583/ijep.2019.3356

Razouki, A., Khzami, S. E., Selmaoui, S., & Agorram, B. (2021). The contribution of physical and sports education to health education of Moroccan middle school students: Representations and practices of teachers. J. Educ. Health Promot. 10(1). https://doi.org/10.4103/jehp.jehp_1021_20

Reeve, J., & Cheon, S. H. (2016). Teachers become more autonomy supportive after they believe it is easy to do. Psychol. Sport Exerc. 22, 178-189. https://doi.org/10.1016/j.psychsport.2015.08.001

Reeve, J., Cheon, S. H., & Yu, T. H. (2020). An autonomy-supportive intervention to develop students’ resilience by boosting agentic engagement. Int. J. Behav. Dev. 44(4), 325-338. https://doi.org/10.1177/0165025420911103

Reuker, S. (2017). The noticing of physical education teachers: a comparison of groups with different expertise. Phys. Educ. Sport Pedagogy. 22(2), 150-170. https://doi.org/10.1080/17408989.2016.1157574

Sanchez, B., Byra, M., & Wallhead, T. L. (2012). Students’ perceptions of the command, practice, and inclusion styles of teaching. Phys. Educ. Sport Pedagogy. 17(3), 317-330. https://doi.org/10.1080/17408989.2012.690864

Sánchez-Oliva, D., Mouratidis, A., Leo, F. M., Chamorro, J. L., Pulido, J. J., & García-Calvo, T. (2020). Understanding physical activity intentions in physical education context: A multi-level analysis from the self-determination theory. Int. J. Environ. Res. Public Health. 17(3), 799. https://doi.org/10.3390/ijerph17030799

Sánchez-Oliva, D., Pulido-González, J. J., Leo, F. M., González-Ponce, I., & García-Calvo, T. (2017). Effects of an intervention with teachers in the physical education context: A Self-Determination Theory approach. PloS one, 12(12), e0189986. https://doi.org/10.1371/journal.pone.0189986

Sanchez-Oliva, D., Sanchez-Miguel, P. A., Leo, F. M., Kinnafick, F. E., & García-Calvo, T. (2014). Physical education lessons and physical activity intentions within Spanish secondary schools: A self-determination perspective. J. Teach. Phys. Educ. 33(2), 232-249. https://doi.org/10.1123/jtpe.2013-0043

Sánchez-Oliva, D., Viladrich, C., Amado, D., González-Ponce, I., & García-Calvo, T. (2014). Prediction of Positive Behaviors in Physical Education: A Self-Determination Theory perspective//Predicción de los comportamientos positivos en educación física: una perspectiva desde la Teoría de la Autodeterminación. Revista de Psicodidáctica, 19(2). 10.1387/RevPsicodidact.7911

Sato, T., & Haegele, J. A. (2017). Graduate students’ practicum experiences instructing students with severe and profound disabilities in physical education. Eur. Phys. Educ. Rev. 23(2), 196-211. https://doi.org/10.1177/1356336X1664271

Sato, T., Haegele, J., Saito, M., & Sawae, Y. (2022). The professional socialisation of Japanese graduate students during adapted physical education practicum experiences. Int. J. Disabil. Dev. Educ. 69(2), 594-608. https://doi.org/10.1080/1034912X.2020.1719049

Schnitzius, M., Kirch, A., Spengler, S., Blaschke, S., & Mess, F. (2021). What makes a physical education teacher? Personal characteristics for physical education development. Br. J. Educ. Psychol. 91(4), 1249-1274. https://doi.org/10.1111/bjep.12415

Schwamberger, B., & Curtner-Smith, M. (2018). Moral development in sport education: A case study of a teaching-oriented preservice teacher. Phys. Educat. 75(3), 546-566. 10.1177/1356336X17753024

Shen, B., McCaughtry, N., Martin, J. J., Fahlman, M., & Garn, A. C. (2012). Urban high-school girls’ sense of relatedness and their engagement in physical education. J. Teach. Phys. Educ. 31(3), 231-245. https://doi.org/10.1123/jtpe.31.3.231

Sinelnikov, O. A., & Hastie, P. (2010). A motivational analysis of a season of Sport Education. Phys. Educ. Sport Pedagogy. 15(1), 55-69. https://doi.org/10.1080/17408980902729362

Sparks, C., Dimmock, J., Lonsdale, C., & Jackson, B. (2016). Modeling indicators and outcomes of students’ perceived teacher relatedness support in high school physical education. Psychol. Sport Exerc. 26, 71-82. https://doi.org/10.1016/j.psychsport.2016.06.004

Sparks, C., Dimmock, J., Whipp, P., Lonsdale, C., & Jackson, B. (2015). “Getting connected”: High school physical education teacher behaviors that facilitate students’ relatedness support perceptions. Sport Exerc. Perform. Psychol. 4(3), 219. 10.1037/spy0000039

Sparks, C., Lonsdale, C., Dimmock, J., & Jackson, B. (2017). An intervention to improve teachers’ interpersonally involving instructional practices in high school physical education: Implications for student relatedness support and in-class experiences. J. Sport Exerc. Psychol. 39(2), 120-133. https://doi.org/10.1123/jsep.2016-0198

Sturm, D. J., Bachner, J., Renninger, D., Haug, S., & Demetriou, Y. (2021). A cluster randomized trial to evaluate need-supportive teaching in physical education on physical activity of sixth-grade girls: A mixed method study. Psychol. Sport Exerc. 54, 101902. https://doi.org/10.1016/j.psychsport.2021.101902

Syrmpas, I., Digelidis, N., & Watt, A. (2016). An examination of Greek physical educators’ implementation and perceptions of Spectrum teaching styles. Eur. Phys. Educ. Rev. 22(2), 201-214. https://doi.org/10.1177/1356336X15598789

Syrmpas, I., Digelidis, N., Watt, A., & Vicars, M. (2017). Physical education teachers' experiences and beliefs of production and reproduction teaching approaches. Teach. Teach. Educ. 66, 184-194. https://doi.org/10.1016/j.tate.2017.04.013

Syrmpas, I., Papaioannou, A., Digelidis, N., Erturan, G., & Byra, M. (2020). Higher-order factors and measurement equivalence of the spectrum of teaching styles’ questionnaire across two cultures. J. Teach. Phys. Educ. 40(2), 245-255. 10.1123/jtpe.2019-0128

Telford, R. M., Olive, L. S., Keegan, R. J., Keegan, S., & Telford, R. D. (2021). Teacher and school outcomes of the Physical Education and Physical Literacy (PEPL) approach: a pragmatic cluster randomised controlled trial of a multicomponent intervention to improve physical literacy in primary schools. Phys. Educ. Sport Pedagogy. 26(1), 79-96. https://doi.org/10.1080/17408989.2020.1799965

Tenório, M. C. M., Tassitano, R. M., Weaver, R. G., & Lima, M. D. C. (2021). Effects of a teacher training intervention on teachers' and students' motivation to physical education class. J. Phys. Educ. 32, e3208. 10.4025/jphyseduc.v32i1.3208

Tessier, D., Sarrazin, P., & Ntoumanis, N. (2010). The effect of an intervention to improve newly qualified teachers’ interpersonal style, students motivation and psychological need satisfaction in sport-based physical education. Contemp. Educ. Psychol. 35(4), 242-253. https://doi.org/10.1016/j.cedpsych.2010.05.005

Tilga, H., Hein, V., Koka, A., & Hagger, M. S. (2020). How physical education teachers’ interpersonal behaviour is related to students’ health-related quality of life. Scand. J. Educ. Res. 64(5), 661-676. https://doi.org/10.1080/00313831.2019.1595718

Tilga, H., Hein, V., Koka, A., Hamilton, K., & Hagger, M. S. (2019). The role of teachers’ controlling behaviour in physical education on adolescents’ health-related quality of life: Test of a conditional process model. Educ. Psychol. 39(7), 862-880. https://doi.org/10.1080/01443410.2018.1546830

Tilga, H., Kalajas-Tilga, H., Hein, V., & Koka, A. (2021). Web-based and face-to-face autonomy-supportive intervention for physical education teachers and students’ experiences. J. Sports Sci. Med. 20(4), 672. https://doi.org/10.52082/jssm.2021.672

Tilga, H., Kalajas-Tilga, H., Hein, V., Raudsepp, L., & Koka, A. (2020). How does perceived autonomy-supportive and controlling behaviour in physical education relate to adolescents' leisure-time physical activity participation? Kinesiology, 52(2), 265-272. 10.26582/k.52.2.13

Todorovich, J. R. (2009). Extremely ego-oriented preservice teachers’ perspectives on teaching physical education. J. Teach. Phys. Educ. 28(2), 155-172.

Tsai, K. C. (2017). Development of the teacher leadership style scale. Soc. Behav. Pers. 45(3), 477-490. 10.2224/sbp.5751

Ullrich-French, S., & Cox, A. E. (2014). Normative and intraindividual changes in physical education motivation across the transition to middle school: A multilevel growth analysis. Sport Exerc. Perform. Psychol. 3(2), 132. 10.1037/spy0000005

Van den Berghe, L., Soenens, B., Vansteenkiste, M., Aelterman, N., Cardon, G., Tallir, I. B., & Haerens, L. (2013). Observed need-supportive and need-thwarting teaching behavior in physical education: Do teachers' motivational orientations matter?. Psychol. Sport Exerc. 14(5), 650-661. https://doi.org/10.1016/j.psychsport.2013.04.006

Van den Berghe, L., Tallir, I. B., Cardon, G., Aelterman, N., & Haerens, L. (2015). Student (dis) engagement and need-supportive teaching behavior: A multi-informant and multilevel approach. J. Sport Exerc. Psychol. 37(4), 353-366. https://doi.org/10.1123/jsep.2014-0150

Van Doren, N., De Cocker, K., De Clerck, T., Vangilbergen, A., Vanderlinde, R., & Haerens, L. (2021). The relation between physical education teachers’(de-) motivating style, students’ motivation, and students’ physical activity: A multilevel approach. Int. J. Environ. Res. Public Health. 18(14), 7457. https://doi.org/10.3390/ijerph18147457

Verma, N., Eklund, R. C., Arthur, C. A., Howle, T. C., & Gibson, A. M. (2019). Transformational teaching, self-presentation motives, and identity in adolescent female physical education. J. Sport Exerc. Psychol. 41(1), 1-9. https://doi.org/10.1123/jsep.2017-0299

Viksi, A., & Tilga, H. (2022). Perceived Physical education teachers’ controlling behaviour and students’ physical activity during leisure time—The dark side of the trans-contextual model of motivation. Behav. Sci. 12(9), 342. https://doi.org/10.3390/bs12090342

Vlachos, O., & Papaioannou, A. G. (2023). Multidimensional Motivational Climate Questionnaire in Physical Education at the Situational Level of Generality (MUMOC-PES). Int. J. Environ. Res. Public Health. 20(5), 4202. https://doi.org/10.3390/ijerph20054202

Webster, C. A., González, S., & Harvey, R. (2012). Physical education teachers’ self-reported communication of content relevance. Phys. Educat. 69(1), 89-103.

Wilkinson, S., Harvey, W. J., Bloom, G. A., Joober, R., & Grizenko, N. (2013). Student teacher experiences in a service-learning project for children with attention-deficit hyperactivity disorder. Phys. Educ. Sport Pedagogy. 18(5), 475-491. https://doi.org/10.1080/17408989.2012.690385

Wright, P. M., & Craig, M. W. (2011). Tool for assessing responsibility-based education (TARE): Instrument development, content validity, and inter-rater reliability. Meas. Phys. Educ. Exerc. Sci. 15(3), 204-219. https://doi.org/10.1080/1091367X.2011.590084

Wright, S., McNeill, M., & Fry, J. M. (2009). The tactical approach to teaching games from teaching, learning and mentoring perspectives. Sport Educ. Soc. 14(2), 223-244. https://doi.org/10.1080/13573320902809153

Yang, C. B., & Dong, M. K. (2017). A Study of the correlation between teachers’ teaching styles and students’ participation motivation in the physical education. J. Balt. Sci. Educ. 16(2), 199. https://doi.org/10.33225/jbse/17.16.199

Zeng, H. Z. (2016). Differences between student teachers' implementation and perceptions of teaching styles. Phys. Educat. 73(2), 285. https://doi.org/10.18666/TPE-2016-V73-I2-6218

Zeng, H. Z., Leung, R., Liu, W., & Hipscher, M. (2009). Physical education in urban high school class settings: Features and correlations between teaching behaviors and learning activities. Phys. Educat. 66(4), 186.

Zhou, Y., Wang, L., Wang, B., & Chen, R. (2022). Physical activity during physical education in elementary school in China: the role of teachers. Phys. Educ. Sport Pedagogy. 27(4), 409-421. https://doi.org/10.1080/17408989.2021.1903410

Zimmermann, J., Tilga, H., Bachner, J., & Demetriou, Y. (2020). The German multi-dimensional perceived autonomy support scale for physical education: Adaption and validation in a sample of lower track secondary school students. Int. J. Environ. Res. Public Health. 17(19), 7353. https://doi.org/10.3390/ijerph17197353
