# Supplementary material for: Leadership practices of physical education teachers and student-related outcomes: a systematic mixed method review and analysis
Source: Front Psychol. 2024 Nov 1;15:1442014. doi: 10.3389/fpsyg.2024.1442014 (PMC11563983; doi:10.3389/fpsyg.2024.1442014)
Supplement: Supplementary file 2 [file Data_Sheet_1.docx]

**Supplementary Figure 1**. Funnel Plot of the Selected Studies to Assess Heterogeneity
